# Biomimetic and bioorthogonal nanozymes for biomedical applications

**DOI:** 10.1186/s40580-023-00390-6

**Published:** 2023-09-11

**Authors:** Changjoon Keum, Cristina-Maria Hirschbiegel, Soham Chakraborty, Soyeong Jin, Youngdo Jeong, Vincent M. Rotello

**Affiliations:** 1 Center for Advanced Biomolecular Recognition, Biomedical Research Division, Korea Institute of Science and Technology (KIST), Seoul, 02792 Republic of Korea; 2 Department of Chemistry, University of Massachusetts, Amherst, 710 North Pleasant Street, Amherst, MA 01003 USA; 3 Department of Chemistry, Hanyang University, Seoul, 04763 Republic of Korea; 4 Department of HY-KIST Bio-Convergence, Hanyang University, Seoul, 04763 Republic of Korea; 5 Division of Bio-Medical Science and Technology, University of Science and Technology, Daejeon, 34113 Republic of Korea

## Abstract

Nanozymes mimic the function of enzymes, which drive essential intracellular chemical reactions that govern biological processes. They efficiently generate or degrade specific biomolecules that can initiate or inhibit biological processes, regulating cellular behaviors. Two approaches for utilizing nanozymes in intracellular chemistry have been reported. Biomimetic catalysis replicates the identical reactions of natural enzymes, and bioorthogonal catalysis enables chemistries inaccessible in cells. Various nanozymes based on nanomaterials and catalytic metals are employed to attain intended specific catalysis in cells either to mimic the enzymatic mechanism and kinetics or expand inaccessible chemistries. Each nanozyme approach has its own intrinsic advantages and limitations, making them complementary for diverse and specific applications. This review summarizes the strategies for intracellular catalysis and applications of biomimetic and bioorthogonal nanozymes, including a discussion of their limitations and future research directions.

## Introduction

As alternatives to natural enzymes, various artificial enzymes, including synthetic peptides [1], molecular catalysts [2], and catalytic nanomaterials [3–5], have been developed for cancer therapy [6], biosensing [7], and diagnostic medicine [8, 9]. Among these artificial enzymes, nanozymes or nanomaterials with catalytic functions have emerged as superior alternatives to natural enzymes owing to their comparable size with protein, which avoids the occurrence of potential steric interference in cellular processes and clearance from cells by diffusion [10]. Nanozymes delivered inside cells have catalyzed chemical reactions that can generate or degrade biomolecules or functional molecules, inducing desired cellular behaviors [11].

Catalysis using nanozymes for intended biochemical cellular reactions can be categorized as biomimetic [12–16], which replicates catalytic functions using natural enzymes, and bioorthogonal [17], which cannot occur in biological processes. Designing catalysts using biomimetic nanozymes (BMNZ) for various applications requires determining the enzymes to be mimicked and the process for implementing their catalytic mechanisms and kinetics. Furthermore, the nanomaterial support for the BMNZ should also be carefully selected considering the intended application. When designing bioorthogonal catalysis using bioorthogonal nanozymes (BONZ), it is necessary to identify the biorthogonal catalysts to be employed for the intended intracellular chemical reactions and their corresponding stabilization mechanisms in the intracellular environments.

The introduction of additional substrates is not necessary when using BMNZs as these nanozymes mostly react with the biomolecules present in the cells. However, the catalytic process and its product molecules might be re-regulated by cellular homeostasis. Unlike BMNZs, BONZs require specific substrates to be added to the cells. However, catalysis using BONZs is independent of the responses of the cells [18]. For both nanozymes, various nanomaterials and catalytic metals with specific features may be used to facilitate intracellular catalysis. However, they might induce undesirable biological effects. Therefore, identifying the advantages and limitations of each catalytic design strategy is critical for the practical use of nanozymes for biomedical applications.

**Fig. 1:**
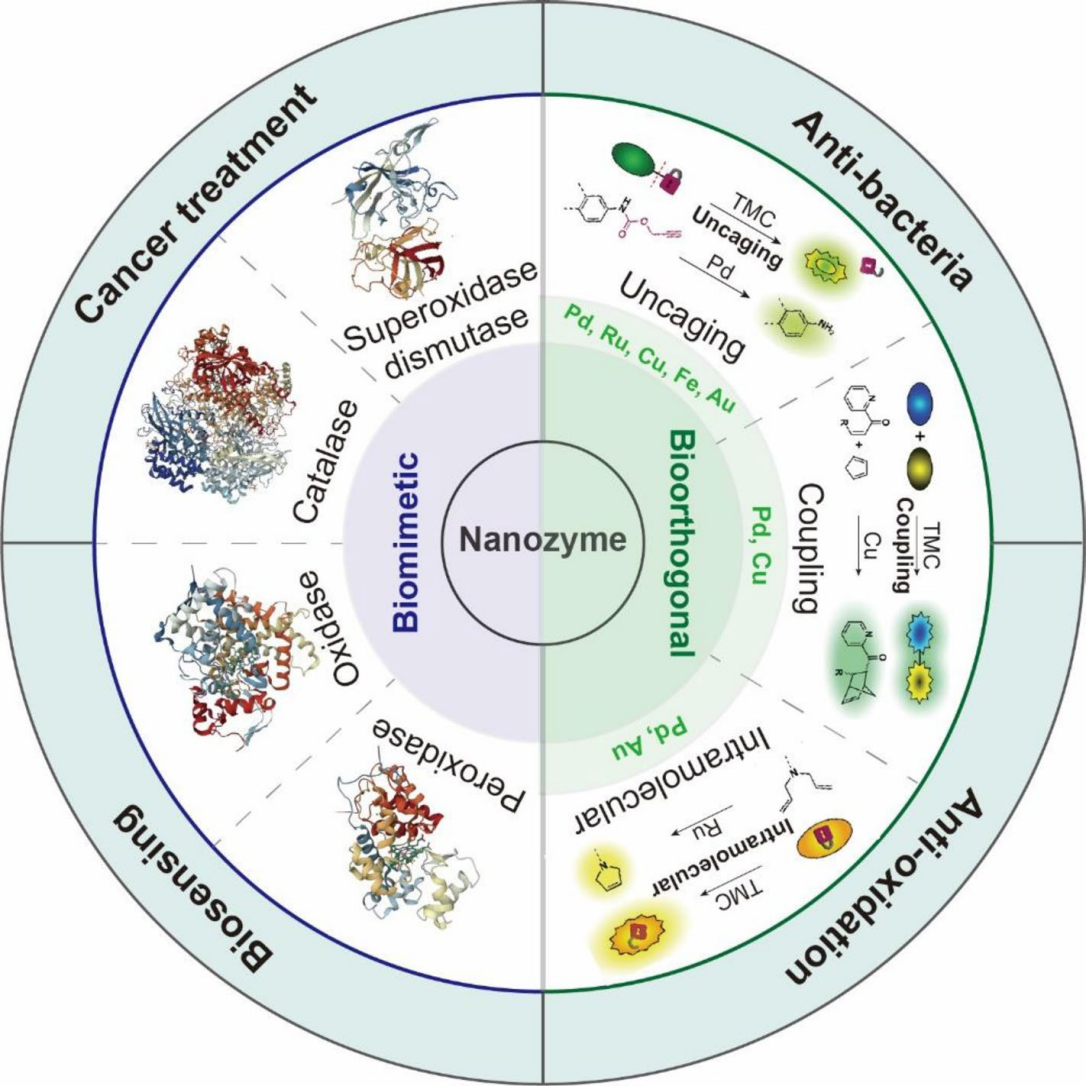
Schematic of the scope of this review on biomimetic and bioorthogonal nanozymes and their biomedical applications

This review provides an overview of the strategies for intracellular catalysis using BMNZs and BONZs, focusing on the mimicry enzymes and nano-supports in BMNZs and the bioorthogonal metal catalysts and chemical reactions in BONZs (Fig. 1). Herein, various biomedical applications of BMNZs are discussed. The allocation of activation or inhibition by chemical or physical cues in BONZs will be described. This review may help in the design and development of nanozymes and their catalysts for regulating intracellular chemical reactions and enzymatic functions in biomedical applications.

## Biomimetic nanozymes (BMNZs)

### Definition of BMNZs

Enzymes are macromolecular biocatalysts produced by proteins or RNA molecules in living organisms [19, 20]. Natural enzymes have been widely used in industrial, medicinal, and biological applications owing to their high catalytic activity and substrate specificity [21]. Despite these promising properties, the application of nanozymes in biosensing, environmental protection, biomedicine, and other fields is limited by their high preparation and purification costs, limited operating stability, highly sensitive catalytic activity to environmental conditions, and recycling and reusing issues [9]. To address these drawbacks, artificial alternatives that mimic natural enzymes have been explored [22].

Since the discovery of the peroxidase-mimetic activity of Fe_3_O_4_ nanoparticles in 2007 [23], numerous studies on nanomaterial-based biomimetic artificial enzymes (termed “nanozymes”) have been published. Nanozymes are nanocatalysts that are used to mimic the activity of native enzymes [24]. Nanozymes demonstrate high stability, facile amplification, and low cost owing to their stable structure and physicochemical properties. BMNZs possess intrinsic enzyme-like activities, including analogs to peroxidase [12], oxidase- [13], catalase [14], superoxide dismutase- [15], and hydrolase activity [16, 25]. The size, shape, structural composition, and surface characteristics of the nanomaterial can affect nanozyme activity and selectivity [5, 26]. For example, numerous studies have consistently shown that nanomaterials with high surface-to-volume ratio exhibit enhanced catalytic activity [25, 26]. Their large surface area exposes more active sites, facilitating the interaction of substrates and catalytic sites. As a result, these high surface area nanocatalysts showed more efficient catalytic activity in various chemical reactions and processes. In addition, the modulation of composition, such as doping of heteroatoms, has proven to be another effective strategy for regulating the activities of nanozymes [27, 28]. In this strategy, the key is that changes in their electronic structures. Finally, the application of additional surface coatings can significantly influence the activities of nanozymes by altering the surface charge and creating a favorable microenvironment for catalysis [29, 30]. Because the versatility of these strategies, BMNZs have been widely used in biosensing, bioimaging, antibacterial, anti-oxidation, medicinal, and environmental protection applications as potential alternatives to natural enzymes [24, 27]. This section introduces the catalytic mechanism and classification of BMNZs are introduced. Furthermore, recent research progress on BMNZ catalysis and its applications in biosensing, bioimaging, and therapeutics is reviewed.

### Types of catalytic reactions using BMNZs

**Fig. 2:**
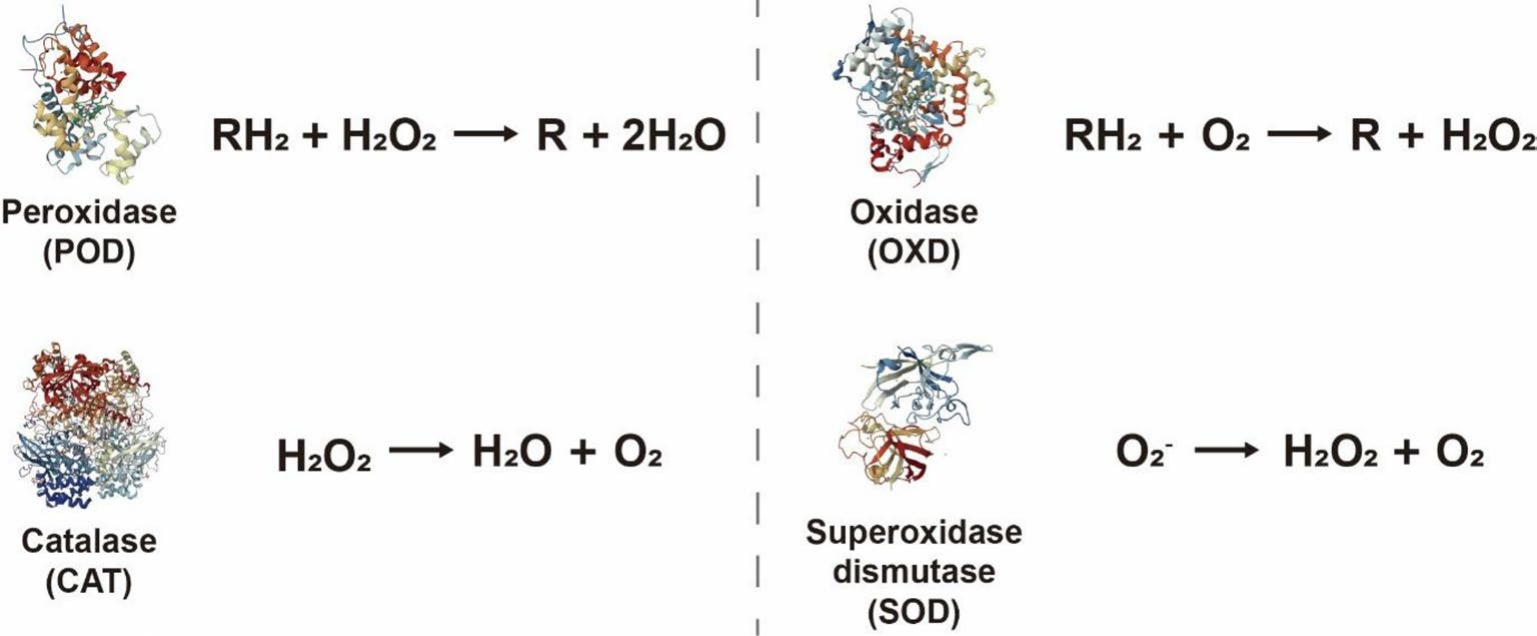
The reaction mechanism of nanozymes mimicking various enzyme reactions

Recently, BMNZs that mimic oxidoreductase enzymes, including peroxidase (POD), oxidase (OXD), catalase (CAT), and superoxide dismutase (SOD), and hydrolytic enzymes have been reported. As discovering new types of BMNZs has been an important aspect of nanozyme research, numerous nanomaterials with enzyme-like properties have been continually developed. However, the catalytic mechanisms and kinetics of these BMNZs remain unclear. This section summarizes the mechanisms and kinetics of catalytic reactions using various BMNZs, classified according to the types of enzymatic activities (Fig. 2).

#### Peroxidase-like activity

Natural PODs stimulate substrate oxidation through the consumption of H_2_O_2_ or organic peroxides [31, 32]. Most natural PODs are ferric heme proteins that can activate H_2_O_2_ to produce highly valent intermediate species, which are capable of abstracting electrons from various substrates. After H_2_O_2_ activation, two-electron oxidation or oxygen atom transfer may occur [33, 34]. Likewise, BMNZs exhibiting POD-like activity also accelerate the oxidation of substrates by catalyzing H_2_O_2_. Additionally, BMNZs with POD-like activities follow the Michaelis–Menten kinetics and ping–pong mechanism. In this mechanism, a catalyst molecule reacts with one substrate to produce one or more products, which are then released, allowing the reaction of another substrate molecule with the enzyme [35]. The catalytic reaction of POD-like BMNZs depends on a reactive oxygen generation pathway to form OH^·^ radicals [36]. The BMNZ splits the O–O bond of the adsorbed H_2_O_2_ molecules on the surface, forming the OH^·^ radicals that can oxidize another substrate. The catalytic mechanisms of noble metal, metal oxide, and single-atom BMNZs with POD-like activities are distinct; they may follow the two-electron oxidation of peroxidase enzyme or one-electron oxidation of H_2_O_2_ through the Fenton reaction. Regardless of the pathway taken, the generated reactive OH^·^ radicals oxidize the organic chemicals as targets [31].

#### Oxidase-like activity

OXD is a natural enzyme that can catalyze the oxidation of substrates at the expense of molecular oxygen and other oxidizing reagents [24]. Similar to that of POD-like BMNZs, the oxidation ability of several OXD-like BMNZs follows the Michaelis–Menten kinetics [13, 37]. The activity of OXD-like BMNZs depends on temperature, pH, and substrates that regulate the electron transfer during the catalytic process. Catalysis using OXD-like BMNZs may proceed either through electron transfer or reactive species production. In the electron transfer pathway, BMNZs receive electrons from the substrate and then transfer them to molecular oxygen through a nucleophilic attack, thereby forming a dioxo intermediate on the active sites of BMNZs [38]. Meanwhile, reactive oxygen species (ROS), such as ^·^O_2_^–^, OH^·^, and ^1^O_2_, which are generated from the molecular oxygen adsorbed on the BMNZ surface, are responsible for the oxidation of substrates in the reactive species production pathway [39].

#### Catalase-like activity

Natural CAT is a common biological catalyst found in all living systems that promote the breakdown of H_2_O_2_ into H_2_O and O_2_. Natural CAT has four Fe-containing heme cofactors, which allows it to bind strongly with the H_2_O_2_ substrate. When CAT reacts with H_2_O_2_, H_2_O_2_ is reduced to H_2_O. Meanwhile, the resting state of the Fe(III) enzyme is oxidized to an O=Fe(IV)– intermediate, which oxidizes a second equivalent of H_2_O_2_ to O_2_, releasing a second equivalent of H_2_O [40]. CAT-like BMNZs follow the Michaelis–Menten kinetics [14]. Their activity is strongly affected by the concentration of the substrates and BMNZs; the rate of H_2_O_2_ decomposition increased with increasing substrate and BMNZ concentrations [41]. In addition, the activity of CAT-like NMNZs is also highly dependent on pH and temperature [9, 42]. Although most favor neutral or alkaline conditions, a few CAT-like BMNZs prefer slightly acidic environments. The underlying catalytic mechanisms of CAT-like BMNZs are poorly understood. However, the known CAT-like mechanisms generally occur through adsorption activation and redox reaction pathways. For example, the catalytic mechanism of metal-based CAT-like BMNZs is based on an adsorption activation process involving free radical chains. In this mechanism, the adsorbed OH species on the metal surfaces causes H_2_O_2_ to favor acid-like breakdown. The decomposed H molecules interact with the preabsorbed OH to produce HO_2_ and H_2_O on the metal surface. Then, the produced HO_2_ transfers H to another H_2_O_2_ molecule, leaving an O_2_ molecule and breaking down H_2_O_2_ to H_2_O and OH [43]. Meanwhile, CeO_2_, which is a known CAT-mimicking BMNZ, follows the redox reaction-dependent pathway [44]. In this mechanism, H_2_O_2_ reduces two Ce^4+^ ions on the surface of CeO_2_ to two Ce^3+^ ions before releasing O_2_ and H^+^. After that, another H_2_O_2_ molecule connects to the two Ce^3+^ sites, thereby splitting the O–O bond to form two H_2_O molecules and regenerate the Ce^4+^ ions. Therefore, the high valence of the active site in BNMZs is favorable to the catalytic process of CAT, similar to natural enzymes.

#### Superoxide dismutase-like activity

SOD is a natural enzyme that catalyzes the dismutation of superoxide radicals, such as HOO^·^ and ^·^O_2_^–^, into O_2_ and H_2_O_2_, providing a key antioxidant defense against oxidative stress in the body. SOD-like BMNZs, such as CeO_2_, Pd, and MnO_2_, are deemed as promising alternatives to SOD owing to their ability to scavenge ^·^O_2_^–^ through the ping–pong mechanism similar to that of natural enzymes. SOD-like BMNZs with unpaired electrons and surface multivalent ions play key roles in catalytic process through adsorption activation and electron transfer. However, the specific mechanism is affected by the architecture and surface ions of BMNZs, as well as the reaction conditions, including pH. For example, the catalytic mechanism on metal- and alloy-based SOD-like BMNZs is related to the protonation of ^·^O_2_^–^ and the adsorption/rearrangement processes of the HO_2_^·^ on the surface. In this process, ^·^O_2_^–^ works as a Brønsted base that captures the proton of H_2_O to form HO_2_^·^ and OH^–^, which are then used to produce H_2_O_2_ and O_2_. The H_2_O_2_ and O_2_ formation reaction is expedited by the high adsorption of HO_2_ on the surface of BMNZs [37]. Meanwhile, nanoceria exhibits SOD-like catalytic activity owing to the reversible valency of the Ce ions and the presence of oxygen vacancies in its structure. On one hand, the oxygen vacancies interact with the superoxide molecules, leading to electron transfer using H_2_O_2_. On the other hand, Ce^4+^ can be reduced to Ce^3+^ through H_2_O_2_ oxidation [44]. On top of these mechanisms, superoxide dismutation through the attraction of electron-deficient regions has also been reported [45].

#### Other enzymatic activities

Hydrolases are ubiquitous enzymes that catalyze the hydrolysis of chemical bonds in biological systems. Although there have been limited studies on their hydrolase-like activity, BMNZs that can mimic hydrolytic enzymes, such as organophosphorus hydrolases, lyase, and carbonic anhydrase, have been reported. There have also been reports about their structures and the mechanism of their enzymatic reactions [46–48]. Inspired by the structure of Zn finger hydrolases, nanomaterials with hydrolase-like activity, such as amino acid coordinated self-assembly, metal–organic frameworks (MOFs), and polymers, have been developed [47, 49, 50]. Generally, similar to hydrolases participating in hydrolytic processes in nature, hydrolase-like BMNZs can facilitate the reaction either by acting as Lewis acid catalysts or by activating H_2_O molecules to produce nucleophilic attack reagents for substrate hydrolysis [51]. Interestingly, most hydrolases contain a Zn active site that is coordinated to the imidazole group of histidine with tetrahedral geometry. These Zn ions can act as nucleophilic reagents or polarize H_2_O molecules for the subsequent hydrolytic processes. Therefore, synthesizing nanostructures that contain Zn^2+^ active site fragments like those in natural enzymes is an efficient method to produce BMNZs with hydrolase-like activity.

### Nanomaterials for BMNZs

**Table:** 

Classification	Materials	Activity	Application	References
Metal-based	Au	POD	Biosensing	[52]
		OXD	Biosensing	[53]
		CAT	Antioxidation	[54]
	Au–NH_2_	CAT	Antioxidation	[55]
	Pt	CAT, SOD	Antioxidation	[56]
	Pd	CAT, SOD	Antioxidation	[57]
	Cu	POD	Biosensing	[58]
	Au@Pt	POD	Biosensing	[59]
		POD, OXD	Biosensing	[60]
	Au–Pt	POD	Biosensing	[61]
Metal oxide-based	Fe_3_O_4_	POD	Cancer therapy	[62]
	Cu_x_O	CAT, SOD	Antioxidation	[63]
		CAT, SOD	Antioxidation	[64]
	MnO_2_	CAT, SOD	Antioxidation	[65]
		POD, OXD	Biosensing	[66]
	Mn_3_O_4_	CAT, SOD	Antioxidation	[67]
	Co_3_O_4_	POD	Biosensing	[68]
		POD, CAT	Biosensing	[69]
	MoO_3_	OXD	Biosensing	[70]
	V_2_O_5_	OXD	Antioxidation	[71]
	CeO_2_	CAT, SOD	Antioxidation	[72]
		CAT, SOD	Antioxidation	[73]
MOF-based	Fe-MOF	POD	Biosensing	[74]
		POD	Antibacterial	[75]
	Cu-MOF	POD	Biosensing	[76]
		SOD	Antioxidation	[77]
		SOD	Biosensing	[78]
	Co-MOF	OXD	Biosensing	[79]
	Ce-MOF	OXD	Biosensing	[80]
		SOD	Antioxidation	[81]
	Pt-MOF	CAT	Cancer therapy	[82]
		CAT, SOD	Antioxidation	[83]
Carbon-based	Graphene oxide	POD	Biosensing	[84]
	Graphene quantum dots	POD	Antibacterial	[85]
	Graphene oxide quantum dots	CAT	Antioxidation	[86]
	Carbon dots	SOD	Antioxidation	[87]
		SOD	Antioxidation	[88]
	FeN_4_-SAzyme	POD	Biosensing	[89]
		POD	Biosensing	[90]
		OXD	Biosensing	[91]
		POD, OXD	Cancer therapy	[92]
		CAT, SOD	Antioxidation	[93]
	FeN_5_-SAzyme	POD	Cancer therapy	[94]
		OXD	Antibacterial	[95]
	ZnN_4_-SAzyme	POD	Antibacterial	[96]
		POD	Cancer therapy	[97]
	CoN_4_-SAzyme	CAT, SOD	Antioxidation	[98]
	CuN_4_-SAzyme	CAT, SOD	Antioxidation	[99]
	PdN_4_-SAzyme	POD, OXD	Cancer therapy	[100]

#### Metal-based BMNZs

Despite their biological inertness, metallic nano-compounds exhibit intrinsic enzymological features similar to those of natural enzymes owing to their distinct structures and properties. Metal-based BMNZs, including metal nanoparticles (NPs) such as Au, Pt, Pd, and Cu NPs and their composites, have been widely used as alternatives for natural enzymes in numerous applications owing to their low cost, biocompatibility, ease of manufacturing, and tunable activity.

**Fig. 3:**
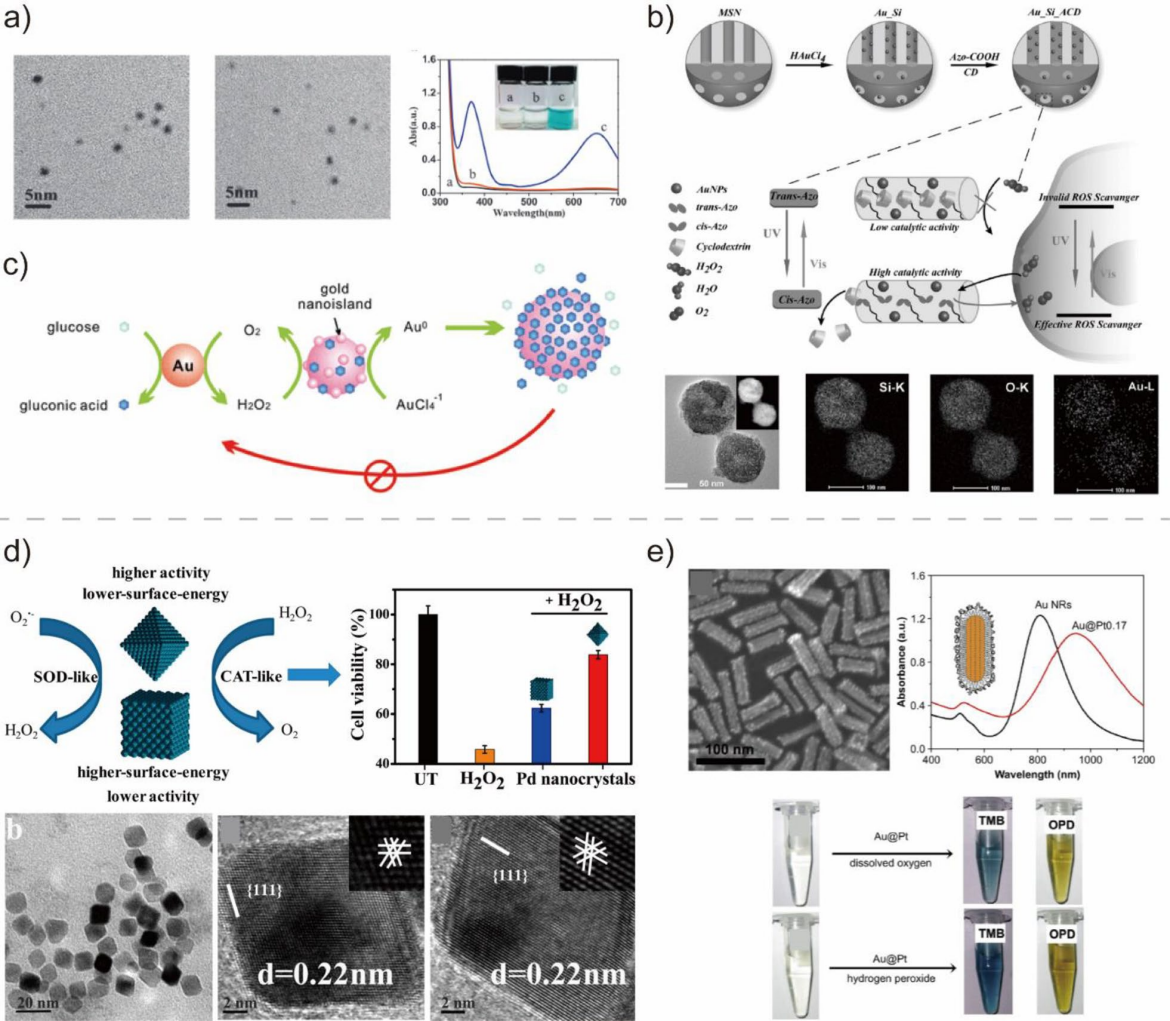
**a** Au NP-based BMNZ that catalyzes the formation of OH^**·**^ radicals. Reprinted from Ref. [101] with permission from Elsevier Inc. Copyright 2015. **b** Synthesis procedure for Au–Si–Azo nanocomposite BMNZ with photo-responsive CAT-like activity. Reprinted from Ref. [54] with permission from Wiley–VCH. Copyright 2017. **c** Schematic of the catalytic mechanism on OXD-mimetic BMNZ composed of Au nanoclusters. Reprinted from Ref. [53] with permission from the American Chemical Society. Copyright 2010. **d** CAT- and SOD-mimetic Pd BMNZ for the reduction of ROS: Schematic of the catalytic mechanism, and TEM and HRTEM images of the Pd octahedrons. Reprinted from Ref. [57] with permission from the American Chemical Society. Copyright 2016. **e** SEM image and UV–vis–NIR absorption spectrum of Au@Pt nanostructure with intrinsic POD and OXD-like activities. Reprinted from Ref. [60] with permission from Elsevier Inc. Copyright 2011

Au NPs have exhibited various intrinsic enzymatic activities, including those of POD, OXD, and CAT [102]. Under acidic conditions, Au NPs can catalyze the formation of OH^·^ radicals from the breaking down of H_2_O_2_ and exhibit POD-like activity (Fig. 3a) [52, 101]. Utilizing the POD-like activity of Au, Jv et al. synthesized positively-charged Au NPs as a catalyst for glucose detection [52]. Au NPs can oxidize 3,3,5,5-tetramethylbenzidine (TMB) using H_2_O_2_. This oxidation reaction produces a blue color in an aqueous solution, which provides a potent colorimetric method to detect glucose by combining glucose oxidase. In addition, Au NPs can also exhibit CAT-like activity at neutral pH conditions. Wang et al. prepared a Au–Si–Azo nanocomposite that enabled the photo-regulation of the CAT-like activity of Au NP-based BMNZs (Fig. 3b) [54]. The Au–Si–Azo composite could reversibly regulate the intracellular concentrations of the ROS and change cell viability according to light exposure. Furthermore, the OXD-like catalytic activity of Au nanoclusters was reported to mimic that of glucose oxidase, which can facilitate reaction with glucose using O_2_ as a co-substrate (Fig. 3c) [53].

Similar to Au NPs, Pt-based NPs can also act as BMNZs and exhibit POD-, OXD-, SOD-, and CAT-like activities. For example, Moglianetti et al. utilized citrate-capped Pt NPs as BMNZs with multifunctional CAT and SOD activities [56]. Pt NPs exhibit catalytic activity comparable to or superior to that of natural enzymes and can restore cellular physiological homeostasis by reducing the ROS levels in an experimental model of cerebral cavernous malformation, which is an oxidative stress-related disorder. Similarly, Ge et al. prepared Pd NPs capable of working as ROS scavengers with SOD- and CAT-like activities (Fig. 3d) [57]. Pd {111} octahedrons have stronger intrinsic antioxidant enzyme-like activity than Pd {100} nano cubes owing to their lower surface energy. Pd NPs reduce the ROS to preserve the homogeneity of mitochondrial membrane potential and attenuate damage to biomolecules, which allows cells to survive oxidative challenges. Cu NPs, which are based on a non-noble metal, exhibit POD-like activity that can facilitate the oxidation reactions of TMB with H_2_O_2_ [58]. Considering the higher activity of Cu NPs than that of natural POD under neutral pH conditions, Cu NPs may be combined with glucose oxidase to develop a colorimetric method for glucose detection.

Integrating two different metals confers synergistic effects in bimetallic NPs that increase their efficiency in biomedical applications, surpassing even that of unary metallic NPs. This suggests the huge potential of bimetallic NPs as BMNZs. He et al. synthesized Au@Pt nanostructures with intrinsic POD- and OXD-like activities (Fig. 3e) [60]. An enzyme-linked immunosorbent assay (ELISA) for mouse interleukin-2 was produced using the Au@Pt nanostructures, which can replace the expensive natural HRP used in conventional ELISAs. In addition to Au@Pt nanostructures, other bimetallic BMNZs, including urchin-like Au@Pt nanohybrids and dumbbell-like Au–Pt NPs, also demonstrated intrinsic POD-like activity, which was applied for the detection of prostate-specific antigen and *E. coli*, respectively [59, 61].

#### Metal oxide-based BMNZs

Metal oxide-based BMNZs are potential artificial enzymes owing to their high surface energy and surface-to-volume ratio [103]. As such, several metal oxide NPs, such as Fe_3_O_4,_ CuO, MnO_2_, Co_3_O_4_, MoO_3_, V_2_O_5_, and CeO_2_, have been reported to exhibit intrinsic enzyme-like activities. Additionally, metal oxide-based BMNZs are promising alternatives to natural enzymes owing to their low cost, high stability, and versatility. Lastly, the low biological toxicity and favorable accumulation of these NPs in biological tissues demonstrate their potential for a wide range of biopharmaceutical applications [9].

**Fig. 4:**
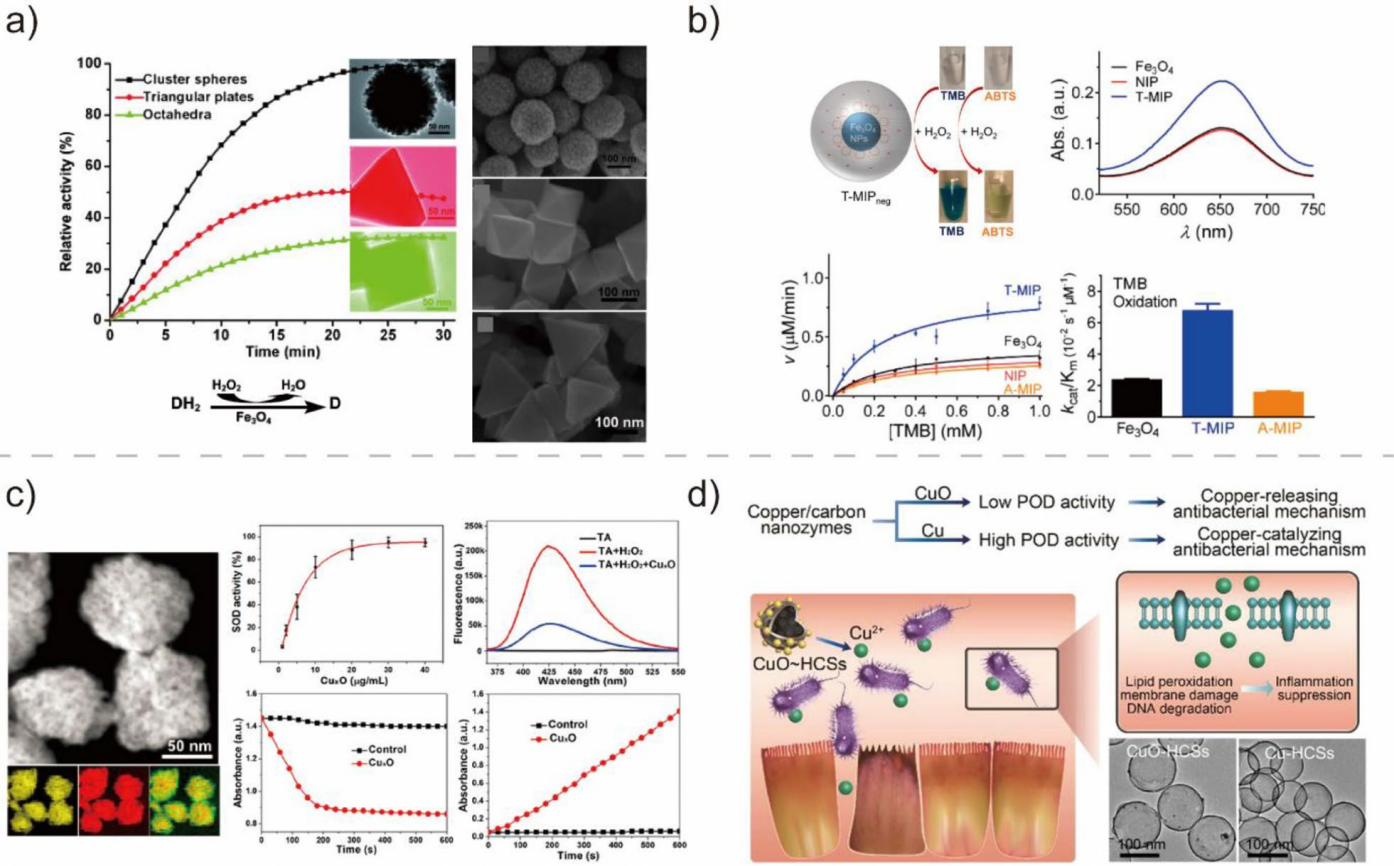
**a** Dependence of the catalytic activity of Fe_3_O_4_-based BMNZs on their structure. Reprinted from Ref. [104] with permission from Wiley–VCH. Copyright 2011. **b** Photographs and schematic of the activity and specificity of molecularly imprinted polymers grown on Fe_3_O_4_ NPs. Reprinted from Ref. [29] with permission from the American Chemical Society. Copyright 2017. **c** TEM image of Cu_x_O nanocluster BMNZs with multifunctional SOD-, CAT-, and POD-like activities. Reprinted from Ref. [63] with permission from the American Chemical Society. Copyright 2019. **d** Synthesis and enzyme-like activities of Cu/C BMNZs with antibacterial activities. Reprinted from Ref. [105] with permission from the American Chemical Society. Copyright 2019

For the POD-like activity of Fe_3_O_4_ NPs, surface Fe^2+^ ions play a key role in the catalytic activation of H_2_O_2_ through the Fenton reaction pathway [23]. Several modification methods were employed to improve the catalytic activity, specificity, and biocompatibility of Fe_3_O_4_ NPs [29, 62, 104, 106, 107]. For example, Liu et al. constructed Fe_3_O_4_ BMNZs with different morphologies, including spherical, octahedral, and triangular plates. The performance of these three Fe_3_O_4_-based BMNZs was compared to study the relationship between the activity and the structure of BMNZs. The POD-like activity of Fe_3_O_4_ followed the order: cluster spheres > triangular plates > octahedrons (Fig. 4a) [104]. In another study, molecularly-imprinted polymers were grown on Fe_3_O_4_ NPs to improve the catalytic selectivity of BMNZs to the target substrate (Fig. 4b) [29]. The synthesized BMNZ exhibited POD-like activity. More importantly, its enzyme selectivity increased by 100-fold owing to the presence of substrate binding pockets in the molecularly imprinted polymers.

Cu_x_O NP-based BMNZs with inherent POD-, CAT-, and SOD-like activities have also been developed. The multi-functionality of Cu_x_O NPs makes them promising for a wide array of biomedical applications, including multi-enzyme mimetics with highly synergistic results. For example, Hao et al. synthesized amino acid-mediated Cu_x_O nanoclusters (NCs) with SOD-, CAT-, and glutathione POD-like activities (Fig. 4c) [63]. The Cu_x_O NC-based BMNZ exhibited exceptional cytoprotective properties against oxidative stress-mediated neurotoxicity in a Parkinson's disease cell-line-based model. In another study, Liu et al. synthesized ultrasmall Cu_5.4_O NPs with similar multifunctional enzymatic capabilities (SOD-, CAT-, and glutathione POD-like activities) that can scavenge ROS and alleviate inflammation-related diseases [64]. Meanwhile, Xi et al. demonstrated that the valency of Cu dictates the enzyme-mimicking activity of CuO NP-based BMNZs. CuO BMNZs containing Cu^2+^ and Cu^0^ exhibited different antibacterial mechanisms that are highly related to their enzyme-like performances (Fig. 4d) [105].

**Fig. 5:**
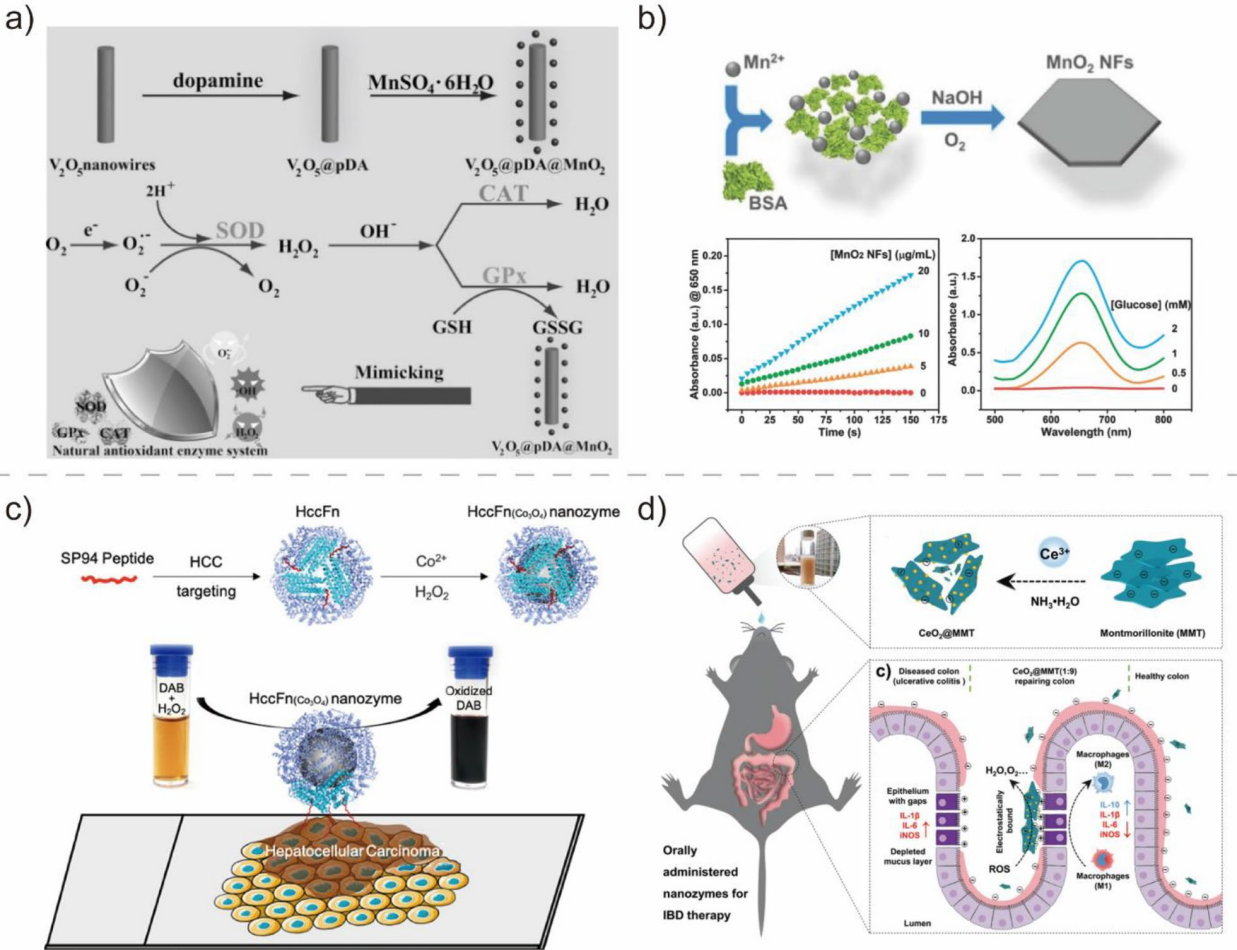
**a** Schematic of the synthesis procedure and ROS scavenging mechanism of V_2_O_5_@pDA@MnO_2_ BMNZ. Reprinted from Ref. [65] with permission from Wiley–VCH. Copyright 2016. **b** Synthesis and TEM images of MnO_2_ nanoflakes tandem BMNZ. Reprinted from Ref. [66] with permission from Wiley–VCH. Copyright 2018. **c** Ferritin nanocage-modified Co_3_O_4_ BMNZ with POD-like activity. Reprinted from Ref. [68] with permission from the American Chemical Society. Copyright 2019. **d** CeO_2_-based BMNZ targeting inflamed colon through scavenging of excessive ROS. Reprinted from Ref. [73] with permission from Wiley–VCH. Copyright 2020

Manganese oxide-based nanomaterials with diverse chemical compositions and multiphase structures, including MnO_2_, MnO, Mn_2_O_3_, Mn_3_O_4_, and MnO_X_ NPs, have been widely used as BMNZs owing to their high catalytic activity, biocompatibility, and biodegradability [67]. Huang et al. developed an antioxidant system composed of MnO_2_ NPs with intracellular antioxidant SOD- and CAT-like activities (Fig. 5a) [65]. Due to the synergistic effects of the multifunctional activities of MnO_2_, the multi-antioxidant system can efficiently catalyze H_2_O_2_ reactions to remove intracellular ROS and protect cell components against oxidative stress. The potential of the MnO_2_-based system in inflammation therapy has also been demonstrated. In another study, Singh et al. developed flower-like Mn_3_O_4_-based BMNZs with SOD-, CAT-, and glutathione POD-like activities [67]; the flower-like BMNZ exhibited a therapeutic potential to prevent ROS-mediated neurological disorders through the redox modulatory effect. Han et al. produced MnO_2_ nanoflakes tandem BMNZ with multi-enzyme mimetic activities (POD and OXD) (Fig. 5b) [66]. Maximizing their multi-functionality, MnO_2_ nanoflakes were used to develop a nonenzymatic one-pot cascade catalysis system for the colorimetric detection of glucose.

BMNZs based on Co, Mo, and V oxides have also received considerable research attention. Among the different Co oxide-based BMNZs, Co_3_O_4_ NPs have been widely used for biomedical applications. For example, Jiang et al. developed ferritin nanocage-modified Co_3_O_4_ BMNZ with POD-like activity through biomineralization (Fig. 5c) [68]. By employing the ferritin nanocage-modified Co_3_O_4_ BMNZs in HCC diagnosis, clinical HCC tissues were successfully distinguished from normal ones. This proved the potential of Co_3_O_4_ BMNZs for biomedical applications. Wang et al. synthesized Co_3_O_4_ nanoplates with pH-dependent POD- and CAT-like activities and fabricated a one-step colorimetric glucose biosensor [69].

Meanwhile, Mo is a transition element that can rapidly change its oxidation state and participate in electron transfer processes during the redox reactions of oxidoreductases [108]. As such, MoO_3_ could possess intrinsic OXD-like activity, which can facilitate the oxidation of ABTS, TMB, and OPD without H_2_O_2_. Using a MoO_3_-based BMNZ, Chen et al. developed a colorimetric method for acid phosphatase detection, which could be applied further in biosensing and clinical diagnostics [70].

In previous reports, Vanadium oxides were also found to possess biomimetic enzymatic activities. For example, V_2_O_5_ nanowires exhibit remarkable antioxidant activity, which can be used in cytoprotective applications by preventing oxidative damage to cellular components through the reduction of H_2_O_2_ in the presence of glutathione [71]. In addition, the performance of Va oxide-based BMNZs with multi-enzymatic activities for biomedical applications has also been reported recently. For example, a BMNZ containing a hexacyanoferrate complex and Va oxide exhibited POD-, SOD-, and CAT-like activities; its POD-like activity was utilized to develop a cascade system for glucose sensing [109]. Ding et al. also utilized the tandem enzyme activity (POD- and OXD-like catalytic properties) of V_2_O_5_ nanobelts for the coulometric detection of glucose under cascade catalysis [110]. In another study, Ma et al. developed a bi-enzyme (POD- and OXD-like activities) synergistic antibacterial system using Va oxide nanodots, which demonstrated excellent in vitro antibacterial performance and in vivo wound healing activity [111].

Similar to other metal oxide-based BMNZs, CeO_2_ NPs as anti-oxidant BMNZs exhibit immense potential for a wide array of biomedical applications owing to their SOD- and CAT-like activities [112, 113]. As CAT- and SOD-mimetic BMNZs, the Ce ions in CeO_2_ nanoparticles can reversibly change from Ce^3+^ to Ce^4+^ states. The presence of both Ce^3+^ and Ce^4+^ states, as well as oxygen vacancies in the lattice of CeO_2_ NPs give them excellent free radical scavenging activity, similar to those of CAT and SOD. In particular, the CAT-like activity of CeO_2_ is usually correlated with the ratio between Ce^3+^ and Ce^4+^. Celardo et al. revealed that CeO_2_ with a low Ce^3+^/Ce^4+^ ratio exhibits CAT-like activity; Ce^4+^ reduction to Ce^3+^ occurs simultaneously with the catalysis of H_2_O_2_ decomposition into dioxygen. In contrast, CeO_2_ NPs with a high Ce^3+^/Ce^4+^ ratio had a SOD-like catalytic activity [44]. Therefore, CeO_2_ NPs may act as dual antioxidant enzyme mimetics for SOD and CAT by adjusting the valence state of Ce ions in their lattice and could be used for the effective regulation of ROS levels. Utilizing these dual antioxidant enzyme activities, Le et al. developed glucose-coated CeO_2_ NPs for the treatment of Alzheimer's disease through neurotoxicity regulation, which involves the formation of ROS [72]. Moreover, CeO_2_ NPs could effectively inhibit Aβ aggregate formation, reduce cellular ROS, and protect cells from Aβ-related neurotoxicity owing to their good biocompatibility. In addition, Zhao et al. utilized the multi-antioxidant enzymatic CAT- and SOD-like activities of CeO_2_ NPs for anti-inflammation therapies for inflammatory bowel disease (Fig. 5d) [73]. When combined with clinically approved montmorillonite and introduced through oral administration, CeO_2_ NPs can effectively reduce inflammation of the colon by scavenging excessive ROS. Moreover, CeO_2_ NPs also demonstrated OXD- and POD-like properties in acidic conditions, catalyzing the production of ROS [114]. Even more, CeO_2_ clusters stabilized on a ZrO_2_ substrate exhibited halo peroxidase-mimicking activities for the catalysis of bromide oxidation with H_2_O_2_, showing impressive antibacterial and anti-biofouling activity [115].

#### MOF-based BMNZs

**Fig. 6:**
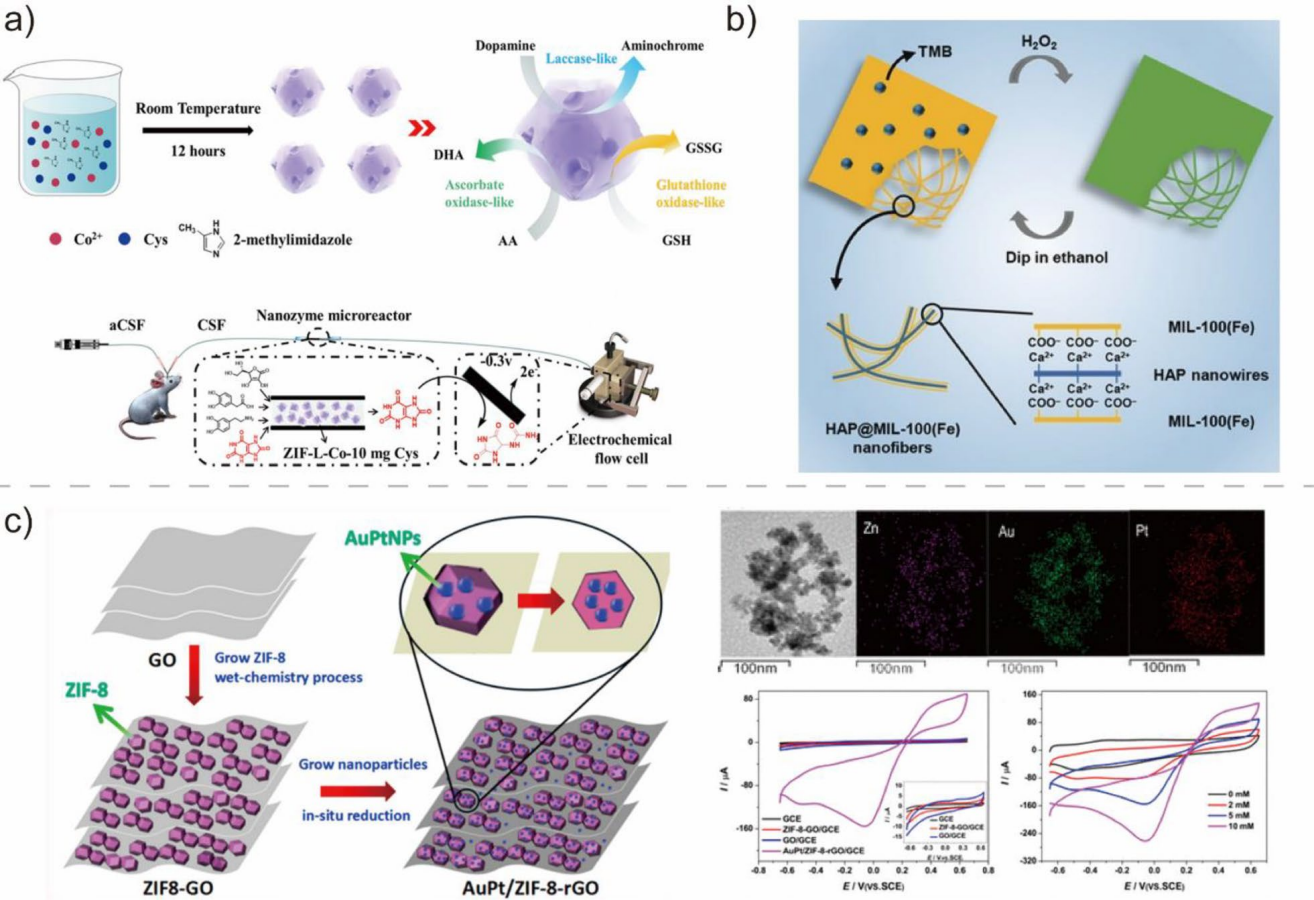
**a** Schematic of the synthesis procedure and properties of MOF-based BMNZ and online measurements of uric acid. Reprinted from Ref. [116] with permission from the American Chemical Society. Copyright 2021. **b** Hydroxyapatite@MIL-100(Fe) core–shell nanofibers with POD-like activity. Reprinted from Ref. [117] with permission from Wiley–VCH. Copyright 2018. **c** AuPt/ZIF-8-rGO BMNZ as a POD mimetic for H_2_O_2_ detection in human serum samples. Reprinted from Ref. [118] with permission from the American Chemical Society. Copyright 2019

MOFs are a special class of porous crystalline materials formed through the self-assembly of a wide range of metal ions or inorganic clusters with organic ligands through coordination bonds under specific conditions [119]. MOFs have attracted considerable research attention owing to their large specific surface area, tunable pore size and shape, ease of modification, and numerous unsaturated metal sites that may serve as active sites during catalysis. The strategic selection of building blocks enables the deliberate tailoring of the structures and functionalities of MOF. As a result, MOFs have emerged as a topical area in materials science and technology and are regarded as versatile materials with potential applications in gas adsorption and separation, storage, catalysis, sensing, and biomedicine [120, 121]. Recently, a few MOF compounds with catalytic activity resembling those of biological enzymes have been discovered. For example, Zr-containing MOFs as organophosphorus hydrolase mimetics for the degradation of organophosphate have been developed [49]. Similar to the conventional metal- and metal oxide-based BMNZs, MOF BMNZs contain metal active centers. However, MOF BMNZs offer the advantage of highly tunable structures and functions, low cost, and easy fabrication. As part of that, introducing defects into the crystal structure of MOFs can finely modify their structure and further endow enzyme-mimetic activity (Fig. 6a) [116]. In this section, recent advancements in the preparation of MOF-based BMNZs are summarized.

Fe-containing MOFs can be used as catalysts for the reaction of H_2_O_2_ with substrates with their POD-like enzymatic activity. For example, Liu et al. developed a nanosized Fe-MIL-88NH_2_ with intrinsic POD-like activity for colorimetric glucose detection [74]. In addition, Chen et al. synthesized hydroxyapatite@MIL-100(Fe) core–shell nanofibers, a Fe-based MOF composite, which exhibited POD-like activity and effectively facilitated the oxidation of various substrates in the presence of H_2_O_2_ (Fig. 6b) [117]. Hydroxyapatite@MIL-100(Fe) was used to develop a colorimetric sensor for H_2_O_2_, glucose, and ascorbic acid. MOF@covalent organic framework (COF) BMNZs have also been developed. Zhang et al. developed high-efficiency POD mimetics based on NH_2_-MIL-88B (Fe) with the Fe node and COF as the active metal center and enzyme binding pockets, respectively. Using this material configuration, a pore microenvironment near the Fe active sites of MOF was formed, allowing the enrichment of TMB and H_2_O_2_ substrates through non-covalent interactions. The MOF@COF BMNZ was used to inhibit bacterial growth and promote wound healing [75]. Other Fe-based MOF BMNZs, including Fe(III)-BTC, MIL-53(Fe), and MIL-101(Fe), have also been reported as POD-mimetic BMNZs [122–124]. In another study, Keum et al. synthesized a Cu-doped zeolitic imidazolate framework-8 (Cu/ZIF-8) [76]. The introduction of Cu ions conferred POD-like enzymatic activity to the pristine ZIF-8. Furthermore, Cu/ZIF-8 facilitated the peroxidation of a fluorescence dye inside a cancer cell through its interaction with H_2_O_2_, allowing cancer cell targeting imaging.

Metals and metal oxide NPs with POD-like activities have also been incorporated into MOF-based BMNZs. For example, Jiang et al. synthesized a Fe_3_O_4_/MIL-101(Fe) BMNZ with POD-like catalytic activity through an ultrasound-assisted electrostatic self-assembly method [125]. Fe_3_O_4_/MIL-101(Fe) had a high saturation magnetization value, making it very responsive to magnetic fields. This catalyst effectively facilitated the dimerization of H_2_O_2_ and *o*-phenylenediamine. In another study, Zhang et al. prepared a AuPt/ZIF-8-rGO BMNZ through the in situ reduction of AuPt nanoparticles (Fig. 6c) [118]. The AuPt/ZIF-8-rGO BMNZ with POD-like catalytic activity was effective for H_2_O_2_ detection in human serum samples owing to the ultra-small size and great dispersion of the AuPt nanoparticles. Meanwhile, Cui et al. prepared Prussian blue NPs on a MOF (PB on MOF) through an in situ growing technique [126]. The synthesized PB on MOF exhibited POD-like properties and can catalyze the oxidation of catechol and TMB in the presence of H_2_O_2_.

**Fig. 7:**
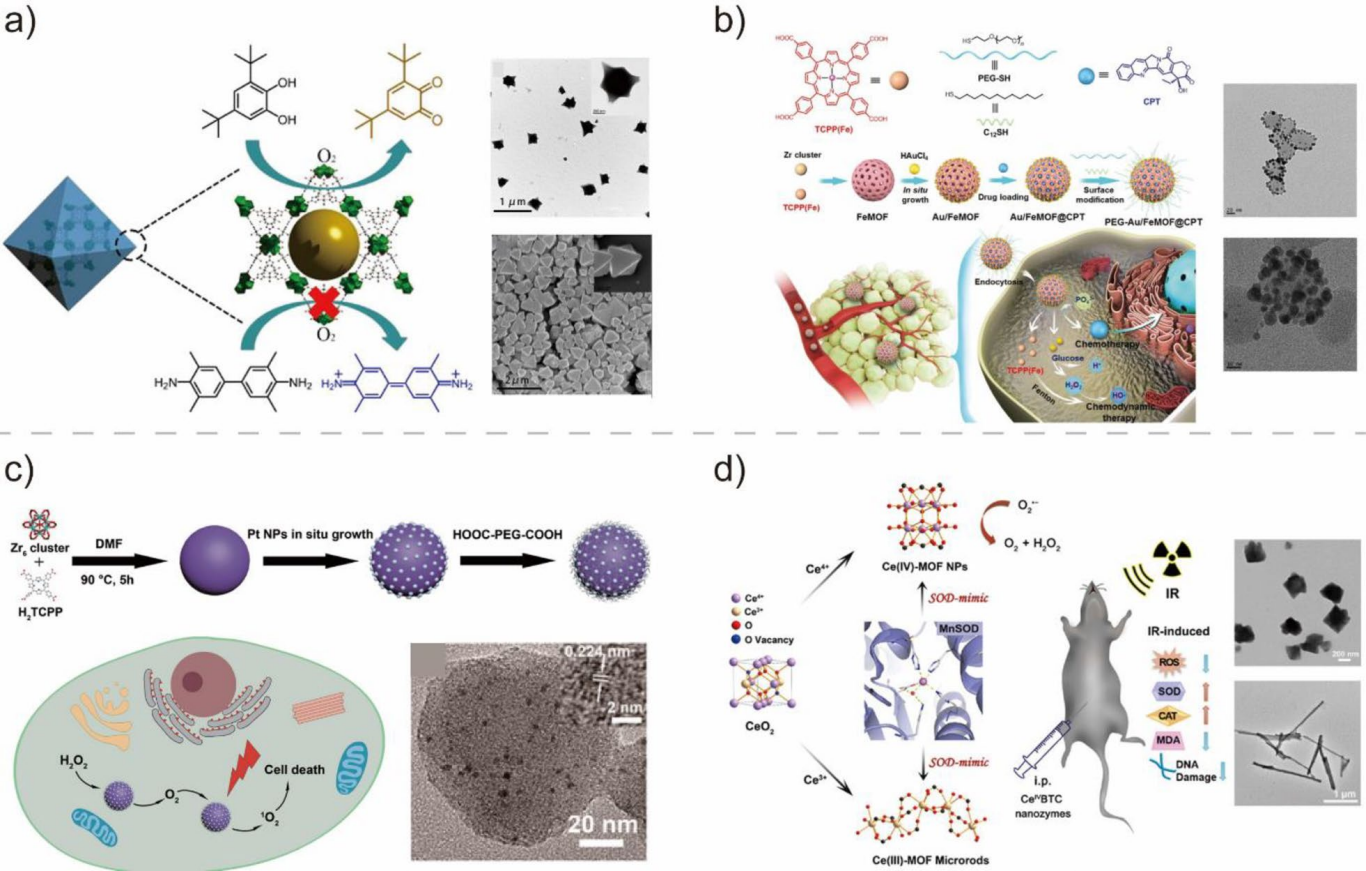
**a** Schematic and SEM images of MOF-818 BMNZ containing trinuclear Cu centers as an OXD mimetic. Reprinted from Ref. [127] with permission from the American Chemical Society. Copyright 2020. **b** Schematic of the synthesis process for the OXD-mimetic Au NPs on Fe-MOF as tumor growth suppressor for chemo/chemo dynamic therapy. Reprinted from Ref. [128] with permission from Wiley–VCH. Copyright 2020. **c** Schematic for the preparation of PCN-224-Pt and its usage for enhanced photodynamic therapy. Reprinted from Ref. [82] with permission from the American Chemical Society. Copyright 2018. **d** Structure of the Ce-based MOFs (Ce^3+^BTC and Ce^4+^BTC) as a SOD-mimetic BNMZ for ionizing radiation protection. Reprinted from Ref. [81] with permission from the American Chemical Society. Copyright 2022

MOF-based BMNZs could also mimic OXD by activating dioxygen to yield ROS, which subsequently oxidizes substrates. Xiong et al. developed a mixed valent Ce-MOF with OXD-like activity through an in situ partial oxidation method [80]. The synthesized Ce-MOF was used for the colorimetric detection of bio thiols in serum samples. Jin et al. also reported the OXD-like activity of ZIF-67, a Co-based MOF [79]. As an OXD-mimetic BMNZ, ZIF-67 can catalyze the oxidation reactions of colorimetric and fluorescent dyes and be used for a fluorescence ‘turn-on’ sensor to detect bio thiols. In another study, a MOF-818 BMNZ containing trinuclear Cu centers exhibited catechol oxidase properties, allowing it to facilitate the oxidation of *o*-diphenol to *o*-quinone effectively (Fig. 7a) [127]. BMNZ composites with OXD-like activity have been also developed through the in situ growth of materials on MOFs. Ding et al. synthesized a BMNZ composed of Au NPs grown in situ on an Fe-MOF, which was used as a nanomedicine and tumor growth suppressor for chemo/chemo dynamic therapy (Fig. 7b) [128]. The hybrid structure of the composite, which prevented the agglomeration of the Au NPs with OXD-like activity, conferred the BMNZ with excellent stability in physiological environment. Lastly, Au NP-decorated Cu-MOF with OXD-like activity has been synthesized through electrodeposition and was used as a sensing platform for nitrite detection [129].

Recently, MOF-based BMNZs with CAT-like activity that can catalyze H_2_O_2_ decomposition have also been prepared through an immobilization method [130]. Zhang et al. reported the CAT-like activity of a Pt-decorated porphyrinic MOF, PCN-224 (Fig. 7c) [82]. This nanoplatform with high CAT-like activity can effectively catalyze the decomposition of H_2_O_2_ in the hypoxic environment of the tumor, facilitating the production of ^1^O_2_ for cancer cell cytotoxicity through photodynamic therapy (PDT). Similarly, Pt@PCN222-Mn with CAT-like activity can scavenge ROS and efficiently protect mice from ROS related inflammatory bowel disease [83]. In another study, Gat et al. decorated a two-dimensional MOF with Pt NPs, yielding a BMNZ with CAT-like activity that can be used for the PDT of cancer cells [131]. A nanohybrid BMNZ was constructed through the in situ growth of Au NPs on the surface of a porphyrinic MOF [132]. The MOF–Au NP hybrid BMNZ with CAT-like activity can oxygenate the tumor microenvironment through H_2_O_2_ activation, increasing the efficiency of O_2_-dependent radiotherapy. Non-noble metal-based MOF composites with CAT-like activity have also been reported. Tang et al. developed MnTCPP-Hf-FA MOF nanoparticles as CAT mimetic BMNZ that can improve radiotherapy effectiveness in hypoxic cancer while inhibiting cancer recurrence [133]. Yin et al. synthesized a MnFe_2_O_4_@MOF core–shell nanostructured BMNZ with efficient photodynamic antitumor therapeutic effect for O_2_ self-sufficient systems [134]. Furthermore, BMNZs based on zeolitic imidazolate framework-8 (ZIF-8) with CAT-like activity, including BSA-MnO_2_/Ce6@ZIF-8 and Ce6/Cyt c@ZIF-8/HA, have also been produced and used for efficient O_2_-dependent therapy of cancer [135, 136].

MOF-based BMNZs with SOD-like activity have also been developed and used in biomedical applications. Similar to CeO_2_-based BMNZs, Ce-based MOFs also exhibit unique Ce^3+^/Ce^4+^ redox properties, conferring them SOD-like activity. For example, Liu et al. developed two monovalent Ce-based MOFs (Ce^3+^BTC and Ce^4+^BTC) and used them to eliminate superoxide radicals for ionizing radiation protection (Fig. 7d) [81]. To mimic the active sites of Cu/Zn–SOD in nature, Zhang et al. developed a bio-inspired CuTCPP MOF nanodots that can functionally and structurally mimic SOD [77]. CuTCPP MOF effectively protected cell components from ROS-mediated damage and reduced the intracellular inflammatory response, which consequently alleviated endotoxemia. Wu et al. prepared a SOD mimetic based on MOF-818, which is analogous to natural Cu/Zn–SOD [78]. The biomimetic structure of MOF-818 resulted to a high SOD-like activity, making it highly suitable as a colorimetric platform for phosphorylated peptide and protein detection. MOF-818 also exhibited excellent SOD-like activity to scavenge ROS and modulate oxidative stress in diabetic chronic wounds [137].

#### Carbon-based BMNZs

**Fig. 8:**
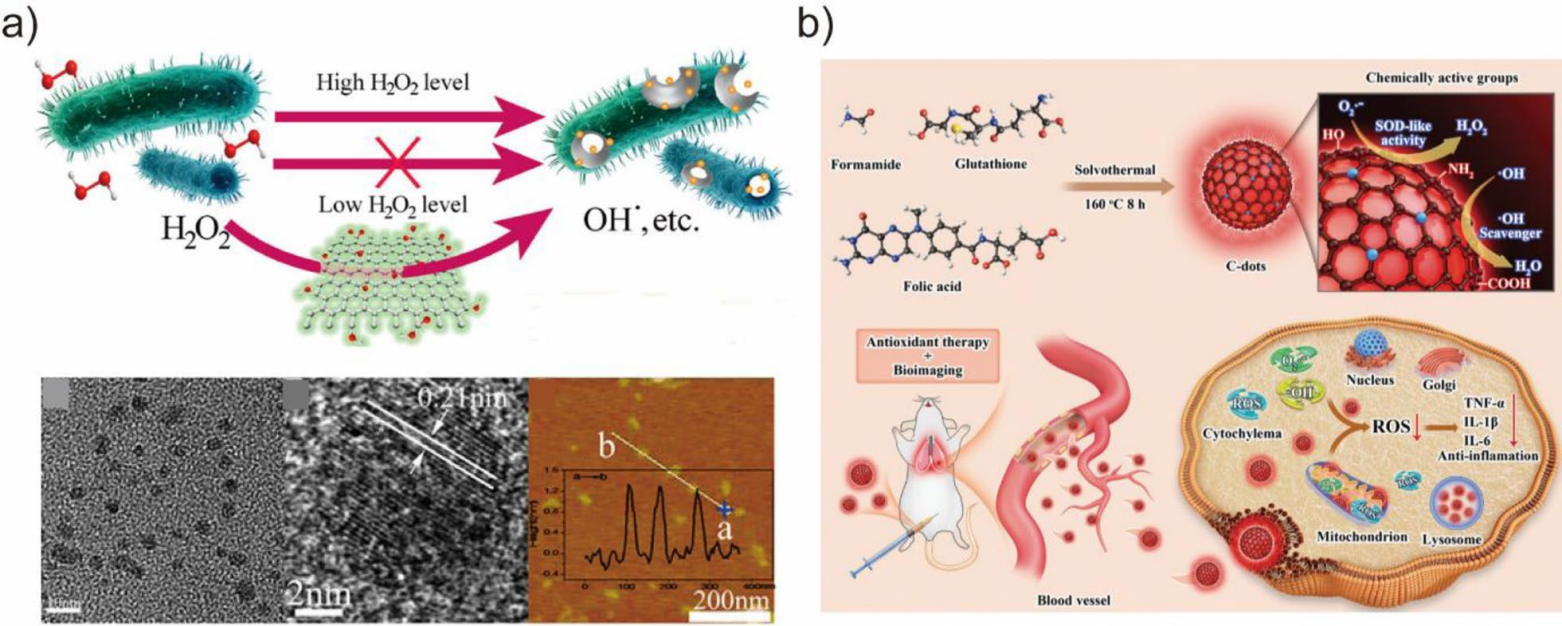
**a** Schematic of antibacterial mechanism and TEM and AFM images of graphene quantum dots. Reprinted from Ref. [85] with permission from the American Chemical Society. Copyright 2014. **b** Schematic of red-emissive carbon dots with SOD-like activity. Reprinted from ref. [88] with permission from Wiley–VCH. Copyright 2023

The performance of various carbon-based nanomaterials, including carbon nanotubes, graphene, fullerenes, graphene quantum dots, and carbon quantum dots, in catalysis, energy, electronic, sensing, and biomedical applications have been evaluated in several studies owing to their excellent physical and chemical properties [138]. In particular, carbon-based nanomaterials have attracted growing interest as metal-free BMNZ catalysts due to their facile preparation, low cost, and robustness against stringent conditions. Sun et al. developed graphene quantum dots with POD-like catalytic activity for the decomposition of H_2_O_2_ and generation of ^·^OH (Fig. 8a) [85]. Reactive ^·^OH radicals exhibit potent antibacterial activity, reducing the need for H_2_O_2_ in wound disinfection. The –C═O and O═C–O– groups in graphene quantum dots act as the catalytically active sites with intrinsic POD-like activity and substrate-binding pockets, respectively [139]. For example, Ma et al. prepared graphdiyne oxide with POD-like activity from a two-dimensional carbon material, graphdiyne [140]. Graphdiyne oxide can catalyze the oxidation of TMB dye, making it an efficient colorimetric sensing platform for the detection of glucose and H_2_O_2_. Song et al. also synthesized carboxyl-modified graphene oxide BMNZ with POD-like activity to develop a selective colorimetric system for glucose detection in diluted blood and fruit juice samples [84]. Fan et al. utilized multifunctional N-doped porous carbon nanospheres for tumor catalytic therapy [141]. The intrinsic multienzyme-like activities of the N-doped porous carbon nanospheres can boost ROS generation in a tumor-specific manner, leading to substantial tumor regression. Zhang et al. also developed a bifunctional metal-free BMNZ based on graphitic carbon nitride, which had OXD- and POD-like activities for glucose and substrate oxidation, respectively [142]. Moreover, the bifunctional cascade catalysis of a graphitic carbon nitride-based has also been demonstrated and used for the real-time colorimetric detection of glucose. The POD-like activity of other carbon-based BMNZs, including single-walled carbon nanotubes [143], carbon nano horns [144], fullerenes [145], has also been reported. Carbon-based CAT- and SOD-mimetic BMNZs have also been developed. Ren et al. reported the CAT-like activity of graphene oxide quantum dots [86]. Due to their biocompatibility and low cytotoxicity, graphene oxide quantum dots facilitated the mitigation of neurotoxicity through antioxidative activities and metabolic regulation. Wu et al. developed poly(ethylene glycolate) hydrophilic carbon clusters with SOD-like activity [146]. Recently, carbon dot-based SOD-mimetic BMNZs were reported [130]. Gao et al. designed and synthesized carbon dots with high SOD-like activity comparable to that of natural enzymes through surface structure tuning [87]. The synthesized carbon dot SOD BMNZ successfully reduced ROS level inside the cells and protected neurons from oxidative stress. In addition, Liu et al. developed red-emissive carbon dots with SOD-like activity (Fig. 8b) [88]. Red-emissive carbon dot BMNZ can efficiently enter cells, scavenge ROS, prevent cellular oxidative damage, and provide treatment effects for acute lung injury owing to their small size, biocompatibility, and antioxidative SOD-like catalytic activity.

Recently, single-atom nanozymes (SAzymes) containing atomic metal active sites on supports have become a new frontier in the development of BMNZ [96]. Among the various types of SAzymes, SAzymes on the carbonaceous supports (M‒NC, M = metals) offer high metallic atom utilization, stability, activity, and selectivity from the M–NX sites in their structure that resemble those in natural metalloenzymes [94, 148]. Due to these characteristics, SAzymes on carbon supports are the best choice for bridging the gap between natural metalloenzymes and BMNZs and introducing new approaches for the development of BMNZs.

**Fig. 9:**
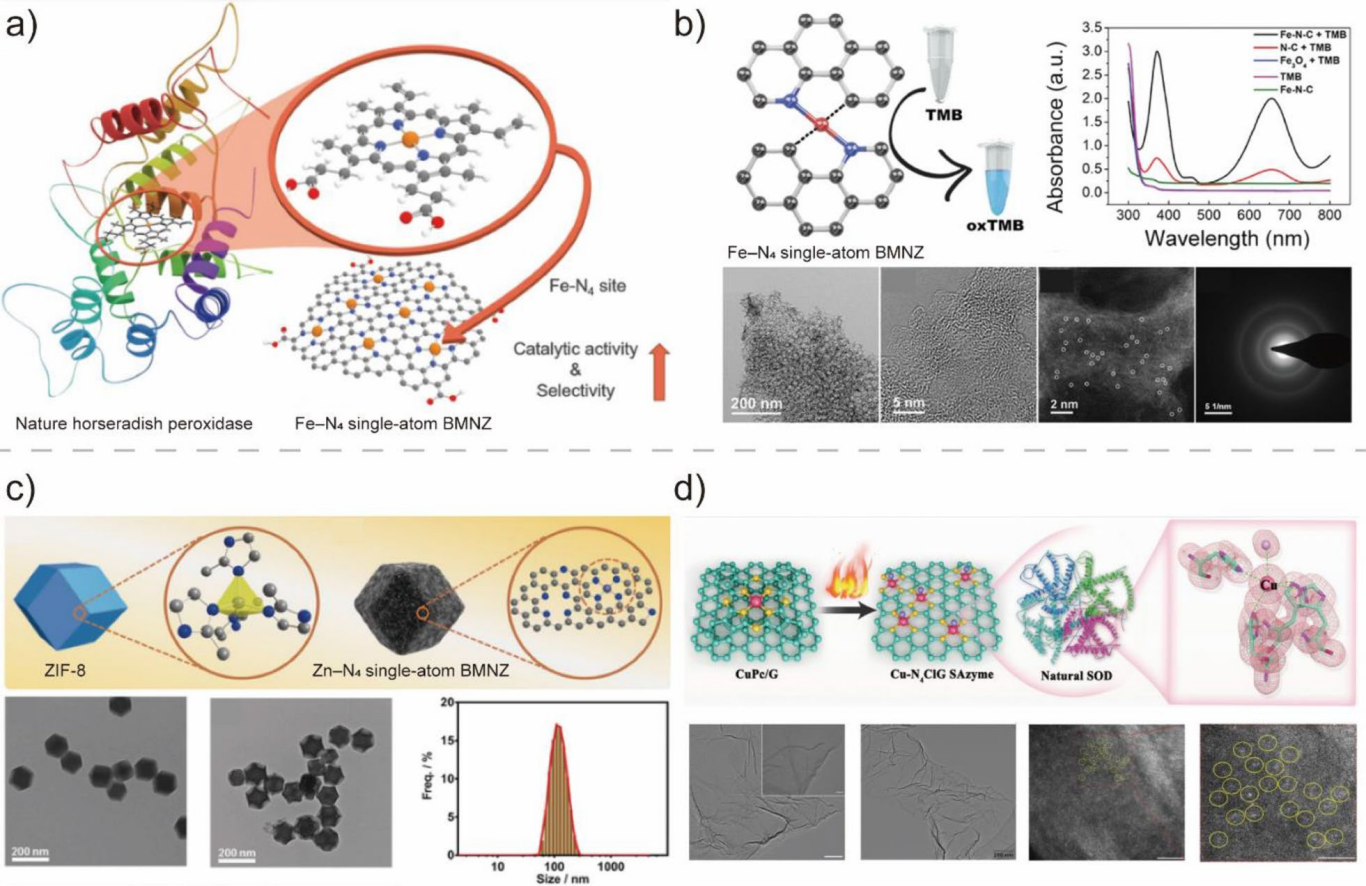
**a** Schematic of the synthesis of Fe–N_4_ single site embedded graphene mimicking the heme cofactor structure of natural horseradish peroxidase (HRP). Reprinted from Ref. [90] with permission from Wiley–VCH. Copyright 2020. **b** OXD-like activity and TEM image of Fe–N_4_ single-atom BMNZ. Reprinted from Ref. [91] with permission from Wiley–VCH. Copyright 2019. **c** Structure and TEM images of the ZIF-8 and ZIF-8 derived Zn–N_4_ single-atom BMNZ. Reprinted from Ref. [96] with permission from Wiley–VCH. Copyright 2019. **d** Schematic and TEM images of Cu–N_4_ single-atom BMNZ with SOD and CAT-like activities. Reprinted from Ref. [147] with permission from Wiley–VCH. Copyright 2023

Fe single-atom embedded N-doped carbon with atomically dispersed Fe–N_X_ structures are similar to the active sites of natural metalloprotease. Therefore, Fe–N_X_ SAzymes can act as BMNZs to achieve enzyme-catalyzed reactions. Inspired by the heme *b* cofactor in natural HRP, Jiao et al. developed atomically dispersed Fe single-atoms on N-doped carbon with densely isolated Fe–N_4_ sites showing high POD-like activities [89]. The synthesized Fe–N_4_ single-atom BMNZ demonstrated highly sensitive and selective detection capability toward intracellular H_2_O_2_. Kim et al. also developed a single-atom BMNZ with POD-like activity based on Fe–N_4_ single-site embedded graphene (Fig. 9a) [90]. Using this BMNZ at bioassay, a trace amount of H_2_O_2_ generated from the evolved acetylcholine in cancer cells was detected with high selectivity and sensitivity. In addition to POD-like activity, single-atom BMNZ based on Fe–N_4_ also exhibited OXD-like activity and can catalyze the transformation of dioxygen into superoxide radical. Exploiting its OXD-like activity, Wu et al. developed a biosensor based on Fe–N_4_ single-atom BMNZ that can evaluate the activity of acetylcholinesterase (Fig. 9b) [91]. Moreover, this bioassay system can monitor trace amounts of organophosphorus compounds, which have been widely used in agriculture as pesticides are responsible for neurotoxicity in humans. Some Fe–N_4_ BMNZs were reported to have multifunctional POD and OXD activities. Zhu et al. developed a Fe–N_4_-centered BMNZ with POD- and OXD-like activities [92]. Owing to its POD-like activity, the Fe–N_4_-centered BMNZ can catalyze the decomposition of H_2_O_2_ into ^·^OH radicals. Meanwhile, it also enabled effective synergistic radio-enzymatic treatment of cancer cells owing to its glutathione OXD-like activity. Indeed, the active sites at natural HRP enzymes have histidine amino acid as the fifth axial ligand to stabilize the active structure and enhance enzyme activity. Xu et al. developed an Fe-based SAzyme with a five-coordinated structure (Fe–N_5_). Fe–N_5_ exhibited superior POD-like activity owing to its optimized coordination structure, which is better than that of the Fe–N_4_ single-atom BMNZ [94]. Fe–N_5_ single-atom BMNZ enabled efficient tumor catalytic therapy in 4T1-tumor-bearing mice. Similarly, Huang et al. synthesized Fe–N_5_ single-atom BMNZ, which catalytically behaved like the axial ligand–coordinated heme cofactor of cytochrome P450 and exhibited superior OXD-like activity relative to that of a square planar Fe–N_4_ BMNZ et al.[95]. This axial ligand coordinated Fe–N_5_ BMNZ was used for efficient wound disinfection in vivo.

Other metal-containing single-atom BMNZs have also been developed as POD-mimetic BMNZs for biomedical applications. For example, Xu et al. developed a ZIF-8 derived Zn–N_4_ active site that can serve as a single-atom BMNZ with excellent POD-like activity et al. (Fig. 9c) [96]. Unsaturated Zn–N_4_ coordinated sites effectively facilitated the decomposition of H_2_O_2_ and formation of ^·^OH radicals, endowing the ZIF-8 derived Zn–N_4_ single-atom BMNZ with high antibacterial activity that promotes wound healing. In addition, MOF-derived flower-like Zn–N_4_ single-atom BMNZ with POD-like activity was also introduced to facilitate ROS-mediated treatment of therapy-resistant tumors [97]. Furthermore, Chang et al. developed a Pd-based single-atom BMNZ with a Pd–N_4_ structure [149]. The optimum atom utilization of the catalytic centers in the Pd–N_4_ single-atom BMNZ resulted to high POD- and glutathione oxidase-like activities, and excellent photothermal conversion performance. Lastly, the Pd–N_4_ single-atom BMNZ also performed well for ferroptosis-boosted photothermal therapy.

Ma et al. developed atomically dispersed bifunctional Fe–N_4_ single-atom BMNZ with CAT- and SOD-like activities [93]. Utilizing its antioxidative activity, Fe–N_4_ BMNZ facilitated the scavenging of ROS to protect the cell from oxidative stress. In addition, Cao et al. reported a Co–N_4_ single-atom BMNZ with multienzyme antioxidative activity for sepsis management [98]. Sepsis is a life-threatening organ dysfunction that causes high morbidity and mortality. Reducing ROS and N levels is critical for sepsis management. The Co–N_4_ single-atom BMNZ efficiently reduced ROS through a tandem reaction of multiple enzymes of SOD, CAT, and glutathione POD, inhibiting proinflammatory cytokine production. Cu–N_4_ single-atom BMNZ also exhibit bifunctional CAT- and SOD-like activities (Fig. 9d) [147]. Zhong et al. developed a graphene-supported Cu–N_4_-centered SAzyme for osteoarthritis treatment. The sequential enzyme mimicking catalytic activity of this SAzyme can protect chondrocytes from oxidative stress-induced apoptosis.

### Various applications of BMNZs

BMNZs are emerging promising alternatives to natural enzymes owing to their comparable catalytic activities. Over the recent years, considerable research progress has been made to improve the properties and catalytic ability of BMNZs, understanding the potential of these natural enzyme mimetics for advanced biomedical applications. Research on BMNZs covered various areas, including developing biomarkers for disease diagnosis, removing ROS for protecting cellular components from toxicity, anti-tumor cancer therapy, and anti-bacterial applications. In this section, research progress on the various biomedical applications of BMNZs are reviewed.

#### BMNZ for cancer treatment and anti-bacterial applications

Cancer is one of the most serious diseases threatening human health globally [152]. Traditional treatments including chemotherapy and surgery usually suffer from unsatisfactory therapeutic efficacy and harmful side effect. To overcome the drawbacks encountered using traditional cancer therapy, BMNZs generating ROS have demonstrated their potential for cancer treatment by targeting features specific to cancer cells, such as acidity, hypoxia, overexpressed hydrogen peroxide, vascular abnormalities, and glucose deprivation. BMNZs for cancer treatments can be generally categorized into two categories based on their ROS generation mechanism, utilizing tumor microenvironment characteristics such as excessive H_2_O_2_ and glucose: (1) BMNZs that produce ROS through POD- and OXD-like catalysis, and (2) BMNZs that yield ROS in the presence of photosensitizers and CAT mimetics in which O_2_ is produced to increase photodynamic therapy efficiency. As result, BMNZs could selectively kill tumors without causing side effects on surrounding normal tissues.

**Fig. 10:**
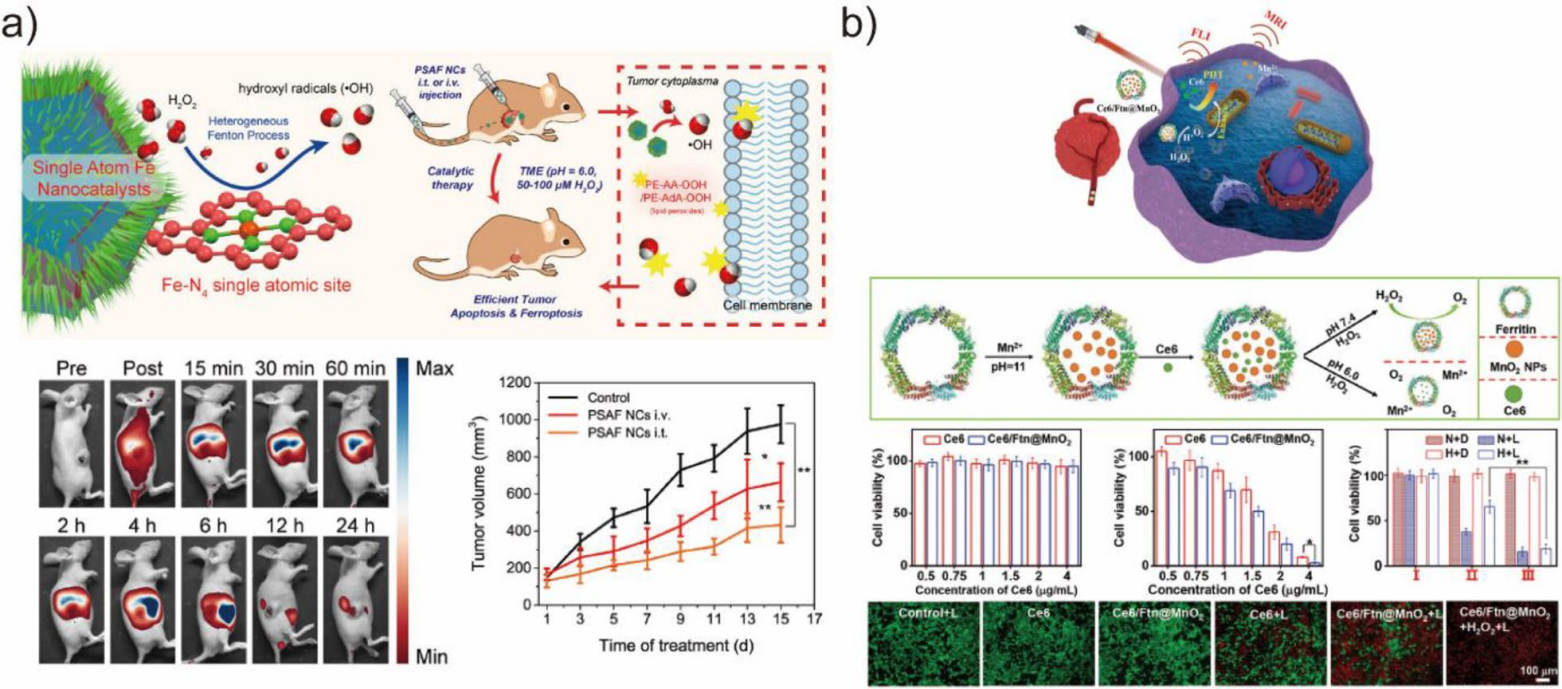
**a** Schematic of Fe-based single-atom BMNZ for catalytic tumor therapy. In vivo biodistribution profile and 4T1 tumor proliferation curves of mice treated with Fe-based single-atom BMNZ. Reprinted from Ref. [150] with permission from the American Chemical Society. Copyright 2019. **b** Schematic of the O_2_ self-supply PDT system constructed using chlorin e6 and MnO_2_ for FLI and MRI bimodal imaging-guided hypoxic cancer treatment and results of in vitro PDT treatment. Reprinted from Ref. [151] with permission from Wiley–VCH. Copyright 2022

Chemodynamic therapy (CDT) is a promising strategy for tumor treatment. In CDT, catalytic reactions are employed to produce highly lethal ^·^OH radicals, which can induce apoptosis in cancerous cells while causing minimal harm to healthy cells [153]. Fe-based POD-mimetic BMNZs are commonly used as CDT agents to initiate the conventional generation of ROS, with catalytic efficiency for ^·^OH generation heavily relying on the levels of reduced Fe^2+^ and H_2_O_2_. For example, Huo et al. developed an Fe-based single-atom BMNZ in which the Fe atoms were isolated in the N-doped carbon support. The Fe-based BMNZ can initiate localized ROS generation for efficient catalytic tumor therapy (Fig. 10a) [150]. In tumors with overexpressed H_2_O_2_ and acidity, Fe-based single-atom BMNZs could produce the toxic ^·^OH radicals through the Fenton reaction for effective and specific inhibition of tumor. Yao et al. also reported triboelectric nanogenerators-stimulated therapeutic catalytic systems that exploited the POD-like activity of ferriporphyrin, which has a structure analogous to that of heme [154]. In this system, the H_2_O_2_ decomposition reaction producing the cytotoxic ^·^OH radicals was accelerated by the human self-generated electric field, thereby improving the therapeutic activity. In addition, BMNZ-based synergistic catalytic chemotherapeutic strategies have also been developed to improve the treatment efficacy. Cai et al. reported improved antitumor effect using a bifunctional Co-based BMNZ [155]. The Co-based single-atom BMNZ exhibited CAT-like activity, decomposing cellular endogenous H_2_O_2_ and generating O_2_. Additionally, it also showed OXD-like properties, converting O_2_ into cytotoxic superoxide radicals and effectively eliminating tumor cells. This sequential process yield considerably improved antitumor effect while minimizing harm to normal tissues.

Photodynamic therapy (PDT) utilizes excitation light, a photosensitizer, and molecular oxygen to generate high cytotoxic singlet oxygen species for the treatment of superficial and localized cancer and other diseases [156]. For example, Wang et al. employed PDT using Ru-based single-atom BMNZ [157]. The single-atom Ru acts as a CAT-like BMNZ, generating oxygen from endogenous H_2_O_2_, improving ROS generation induced by chlorin e6 photosensitizer, and finally causing apoptotic cell death effectively. Similarly, Zhu et al. constructed an O_2_ self-supply PDT system using chlorin e6 and MnO_2_ (Fig. 10b) [151]. The PDT system initiated the breakdown of naturally occurring H_2_O_2_ at the tumor site, generating additional O_2_. This process helped alleviate tumor hypoxia, which is linked to the resistance of the tumors to PDT. Meanwhile, Chang et al. developed a BMNZ-based PDT capable of operating under mild temperature conditions [149]. Therein, a Pd-based single-atom BMNZ with dual POD and OXD-like activities and excellent photothermal conversion performance was employed, leading to ferroptosis through the catalysis of ROS generation and enabling mild PTT.

Bacterial infection is a growing concern [21]. At present, antibiotics are the most widely used treatment model. However, the excessive and improper use of antibiotics has led to significant drug resistance, which has resulted in compromised treatment outcomes and increased mortality rates. Additionally, the formation of biofilms on biological and non-biological surfaces creates an extracellular matrix that acts as a potential barrier for antibiotic penetration, making bacterial infections persistent and exacerbating the challenge of combating microbial infections. Therefore, developing alternative antibacterial agents that avoid bacterial resistance is crucial. As with cancer treatment, BMNZs with POD- and OXD-like activities offer a unique potential for antibacterial applications by generating ROS from O_2_ or H_2_O_2_. The preparation of BMNZs with bacterial selectivity through rational design is of great significance. To date, considerable research progress has been made for BMNZs as a new generation of antibiotics.

**Fig. 11:**
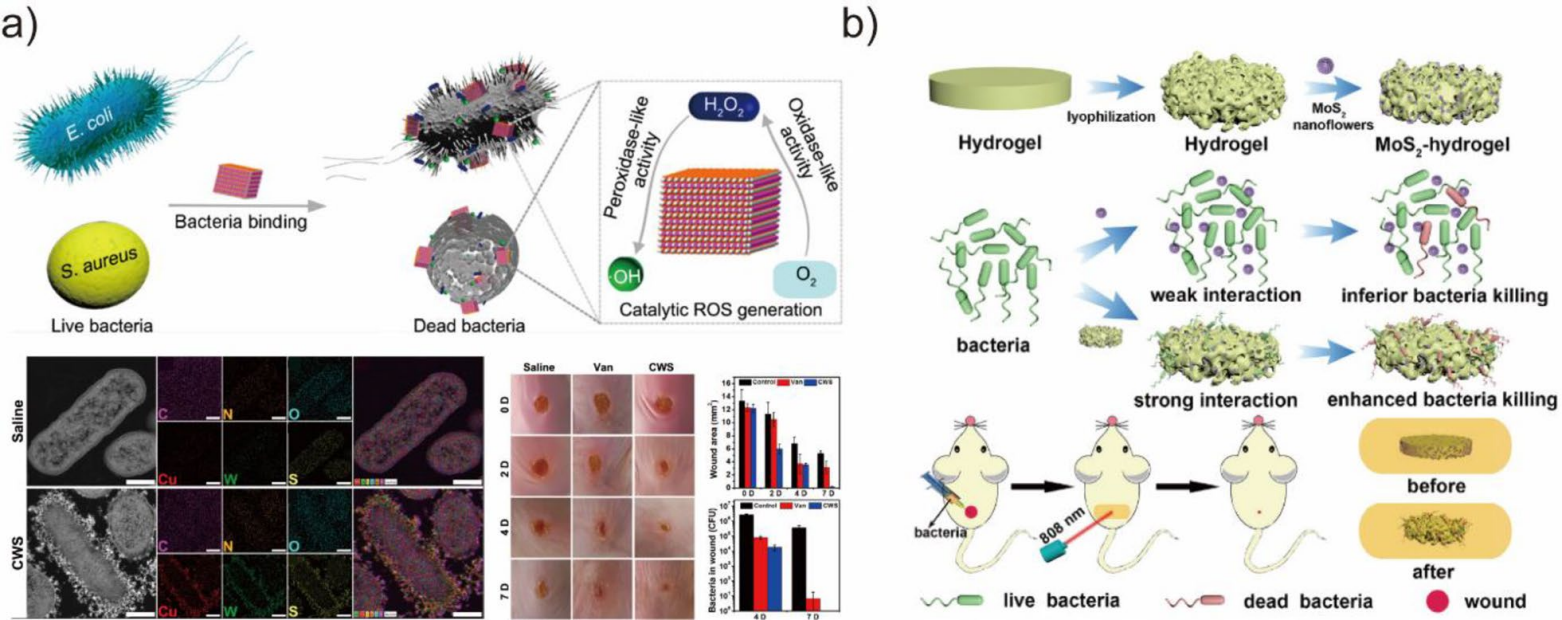
**a** Schematic of the antibacterial mechanism on Cu_2_WS_4_ BMNZ, elemental maps of E. coli cells exposed to Cu_2_WS_4_ BMNZ, and treatment of infected wounds by Cu_2_WS_4_ BMNZ. Reprinted from Ref. [158] with permission from the American Chemical Society. Copyright 2019. **b** Schematic of the fabrication and application for the treatment of wound infection of MoS_2_ BMNZ-hydrogel. Reprinted from Ref. [159] with permission from Wiley–VCH. Copyright 2019

Hu et al. reported the antibacterial property of ultrasmall Au NPs grown on MOFs [160]. The prepared hybrid BMNZ with POD-like activity promoted the production of toxic ^·^OH, which had effective antibacterial activity against Gram-positive and Gram-negative bacteria at low concentrations of H_2_O_2_. The antibacterial activity of Cu_2_WS_4_ BMNZ has also been reported (Fig. 11a) [158]. Cu_2_WS_4_ BMNZ exhibited characteristics similar to those of POD and OXD enzymes, which enabled it to bind selectively to bacteria and stimulate the generation of ROS, thereby eliminating bacteria. The ability to selectively bind to bacteria is crucial in enhancing the antibacterial efficacy of Cu_2_WS_4_ BMNZ and reducing its toxicity toward healthy cells. Sang et al. devised a hydrogel system using BMNZ to address the insufficient interaction between BMNZ and bacteria. Therein, the lifetime and diffusion distance of ROS, which compromises the ability of the BMNZ to kill bacteria, was limited (Fig. 11b) [159]. By leveraging the near-infrared photothermal property and POD-mimicking activity of MoS_2_, the BMNZ-hydrogel was able to efficiently capture bacteria and exhibited superior antibacterial efficacy compared to traditional BMNZ-based antibacterial systems. Moreover, the BMNZ-hydrogel helped reduce inflammation and accelerated wound healing by eliminating dead bacteria from the wound site. Despite the considerable advancements on their usage as antibacterial agents, the non-specificity of ROS in differentiating bacterial and mammalian cells has hindered the development of selective BMNZ-based antimicrobials. To address this issue, Gao et al. created OXD-mimetic AgPd_0.38_ BMNZs that produce ROS on their surface and selectively target bacteria while leaving mammalian cells unharmed [161]. The ROS produced by these BMNZs were bound to the surface. Endocytosis was present in mammalian cells but not in bacteria, unexpectedly acting as an antidote. As a result, these BMNZs have a huge potential to efficiently eliminate antibiotic-resistant bacteria.

#### BMNZ for antioxidation

Excessive levels of ROS can have harmful effects on cells. When ROS are overexpressed, they can cause oxidative damage to lipids, proteins, DNA, and other biological molecules, as well as activate caspase to induce cell apoptosis. Moreover, ROS have been linked to numerous pathological conditions, including cancer, diabetes, neurodegeneration, atherosclerosis, arthritis, and kidney diseases. On top of that, ROS are directly linked to human diseases, aging, and mortality, posing a serious threat to human health and well-being. Therefore, maintaining intracellular redox homeostasis by regulating ROS levels is critical. Antioxidant enzymes, including CAT, SOD, glutathione POD, and peroxiredoxin, play crucial roles in balancing cellular redox levels. However, under pathological conditions, excessive ROS can impair the activity of these enzymes, making intracellular antioxidation mechanisms ineffective in counteracting the overproduction of ROS. BMNZs with natural enzyme-like catalytic activities can be used to regulate ROS generation that protects cells and promotes growth, making them as potential antioxidants.

**Fig. 12:**
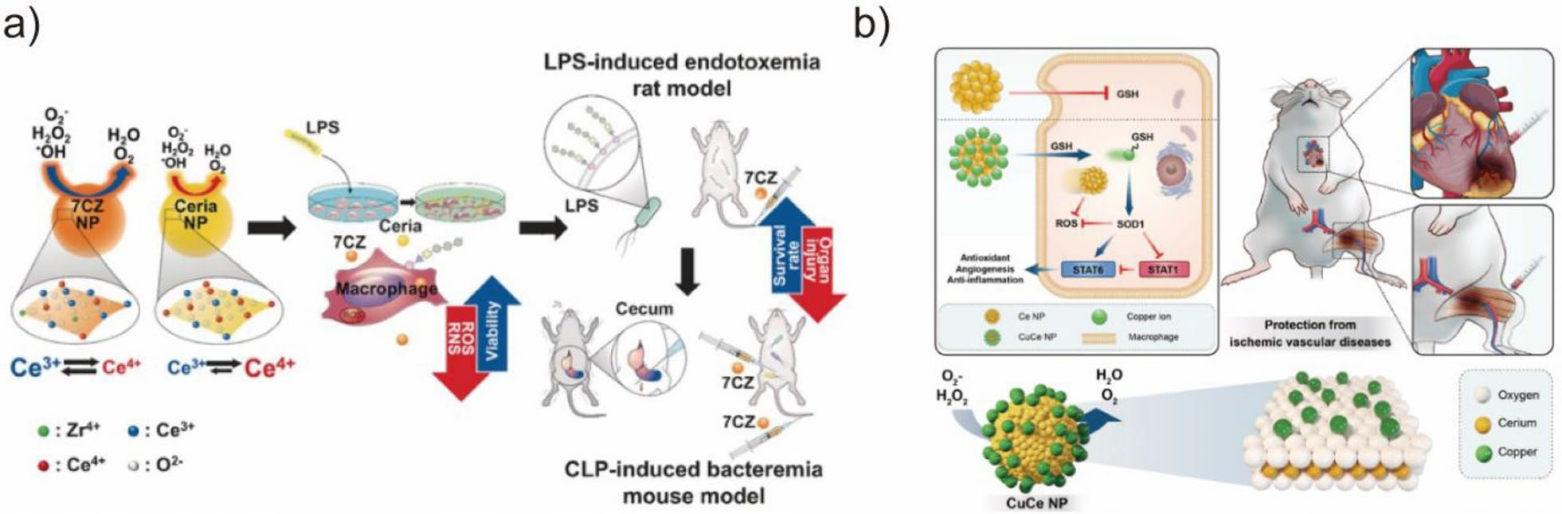
**a** Schematic of CeO_2_ and CeO_2_–ZrO_2_ NPs as therapeutic nanomedicines in sepsis model. Reprinted from Ref. [162] with permission from Wiley–VCH. Copyright 2017. **b** Schematic of CuCe NP-based BMNZ for the protection from ischemic vascular diseases. Reprinted from Ref. [163] with permission from Wiley–VCH. Copyright 2023

CeO_2_ NPs are well-known for their antioxidant properties. Soh et al. synthesized CeO_2_–ZrO_2_ NPs with SOD- and CAT-like activities (Fig. 12a) [162]. The CeO_2_–ZrO_2_ NPs were effective at reducing mortality and inflammatory responses in sepsis by acting as a potent ROS scavenger. Singh et al. prepared CeVO_4_ BMNZ that can regulate ATP levels in neuronal cells under oxidative stress [164]. Furthermore, the CeVO_4_-based BMNZs catalyzed the conversion of superoxide to H_2_O_2_ and O_2_, mimicking the function of SOD1 and SOD2 enzymes without affecting the cellular antioxidant machinery. In another study, Mn_3_O_4_ BMNZ with multi-enzymatic activities (SOD, CAT, and glutathione POD) has been used for the cytoprotection of human cells in a Parkinson’s disease model [67]. The redox modulatory effect of Mn_3_O_4_ controlled the cellular ROS levels, maintaining the redox homeostasis and protecting the cells from cytotoxicity in Parkinson’s disease. Im et al. developed CuCe NP-based BMNZ in which the Ce atoms with the SOD- and CAT-like activities served as antioxidants and allowed the Cu ions to be released for synergetic antioxidant therapy (Fig. 12b) [163]. This synergistic antioxidant BMNZ could scavenge ROS and promote anti-inflammation. Kwon et al. introduced triphenyl phosphonium (TPP) moieties into CeO_2_ NPs and used them for the treatment of Alzheimer's disease [165]. The resulting BMNZ can accumulate in mitochondria, where it can effectively eliminate harmful ROS and prevent neuronal death in an Alzheimer's disease mouse model. Furthermore, the TPP-modified CeO_2_ NPs can mitigate reactive neuroglia and mitochondrial damage. The study suggests that antioxidant BMNZs could be used in mitochondrial therapy for neuroinflammation, with potential applications in the treatment of Alzheimer's disease and other neurodegenerative disorders. Similarly, Hu et al. also developed TPP–MoS_2_ quantum dots with the ability to scavenge ROS and specifically target microglial mitochondria [166]. The TPP–MoS_2_ quantum dots could successfully cross the blood–brain barrier and reduce Aβ-mediated neurotoxicity, thereby suppressing neuroinflammation and treating Alzheimer's disease in mice.

#### BMNZ for biosensing applications

**Fig. 13:**
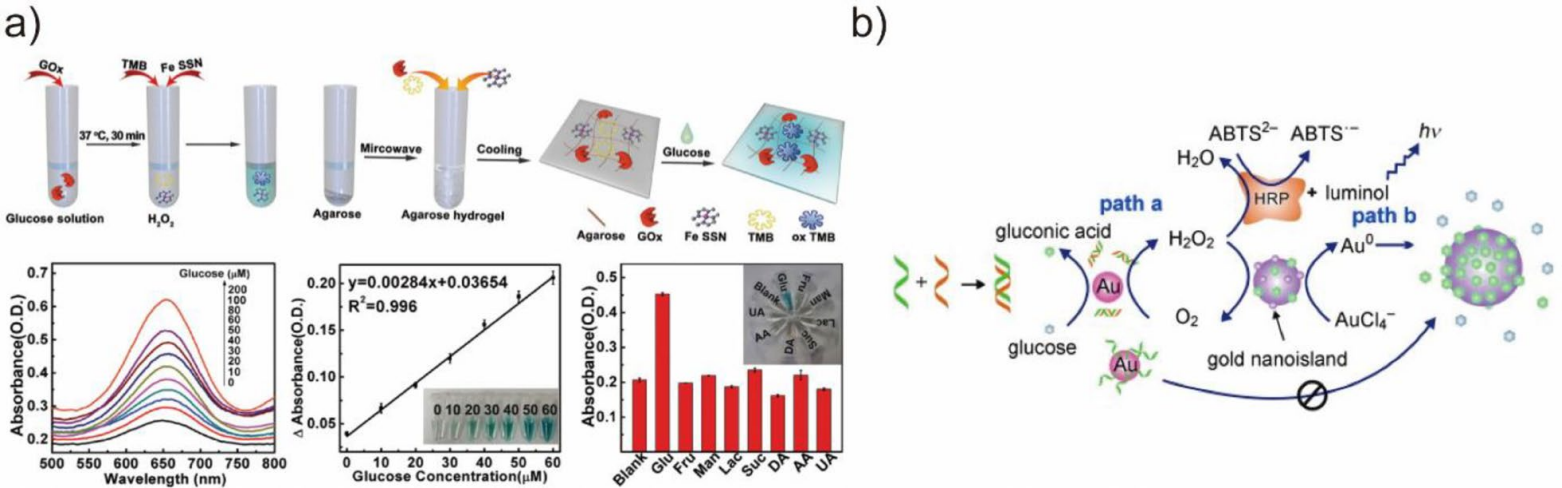
**a** Biocatalytic cascade system composed of glucose oxidase and a single Fe site BMNZ with POD-like for the colorimetric detection of glucose. Reprinted from Ref. [167] with permission from Wiley–VCH. Copyright 2020. **b** Schematic of the operation of a plasmonic probe capable of detection DNA hybridization by the utilizing glucose OXD-mimetic activity of Au NPs. Reprinted from Ref. [12] with permission from Wiley–VCH. Copyright 2011

BMNZs have also been successfully used in biosensing. In particular, they have been employed for the detection of important biological targets, including small molecules like H_2_O_2_ and oxidase substrates, proteins, nucleic acids, and ions [24]. Traditional methods are mainly based on the colorimetric detection using natural HRP enzyme. Despite the high catalytic activity and selectivity of HRP, its inherent drawbacks limit its potential applications. Therefore, since the first report of a colorimetric assay for H_2_O_2_ using Fe_3_O_4_ by Wei et al., BMNZs with POD-like activities have received considerable attention as alternatives for natural HRP for colorimetric detection [168]. Oxidase substrates, such as glucose, can also be detected when oxidase and BMNZs with POD-like activity are utilized. For example, Chen et al. developed a biocatalytic cascade system composed of a single Fe site BMNZ with POD-like activity and glucose oxidase, which was effective in the quantitative detection of glucose (Fig. 13a) [167]. Wu et al. also reported a three-enzyme-based cascade reaction system consisting of POD-mimetic Cu single-atom BMNZ, natural acetylcholinesterase, and choline oxidase. This system could detect trace amounts of acetylcholine, which is an important neurotransmitter in the body [169]. The abnormal expression of glutathione can cause several diseases, such as inflammation and cancer. Considering this, Zhang et al. developed a Ru-based cluster BMNZ with POD- and OXD-like activities that demonstrated efficient OXD-like activity toward O_2_ reduction, which was particularly useful for glutathione detection [170].

For protein detection, Duan et al. synthesized a Fe_3_O_4_-based BMNZ with POD-like activity and used it as an immunochromatographic strip for the detection of the glycoprotein of the Ebola virus [171]. The Fe_3_O_4_ BMNZ could catalyze the oxidation reaction of POD, amplifying the signal and increasing the detection sensitivity by a 100-fold. In another study, Xia et al. synthesized Pd–Ir nanocubes as POD-mimetic BMNZs, which exhibited a 400-fold higher efficiency than that of natural HRP [172]. The colorimetric ELISA prepared using the Pd–Ir nanocubes successfully detected human PSA with a detection limit as low as sub-picogram per milliliter. Several studies have demonstrated the successful detection of nucleic acid using BMNZs. For example, Wang et al. developed a ferric porphyrin and streptavidin-functionalized graphene as a POD-mimetic BMNZ [173]. In the presence of DNA, the structure of the biotinylated molecular beacon could be opened, followed by the binding of streptavidin-functionalized BMNZ. Then, BMNZ catalyzed the oxidation of *o*-phenylenediamine in the presence of H_2_O_2_, producing quantitative electrochemical signals of DNA down to attomolar levels. In addition, Zheng et al. developed a novel plasmonic probe that can detect DNA hybridization by utilizing the glucose OXD-mimetic activity of Au NPs (Fig. 13b) [12]. The developed plasmonic assay avoids the usage of labeled DNA probes for the detection of DNA hybridization, as Au NPs have a high affinity toward single-stranded DNA and the assembling of ssDNA on their surface reduces their glucose oxidase-like activity. Recently, Broto et al. developed CRISPR-based diagnostics to enable the specific sensing of RNA biomarkers [174]. The combination of CRISPR–Cas system-based reaction and BMNZ-linked immunosorbent assay allows the quantitative readout of Cas13-mediated RNA detection using the POD-like activity of Pt@Au BMNZ. The system was successfully used to identify acute myocardial infarction patients. In addition to the above-mentioned targets, BMNZs could also be used to detect ions, such as Cu^2+^ [175], Ag^+^ [176], F^–^ [177], CN^–^ [178], and S^2–^ [179].

## Bioorthogonal nanozymes (BONZs)

Bioorthogonal chemistry describes reactions that do not occur in native living systems. These transformations can be designed to occur dependently without disturbing the native intra- and extracellular processes and specific toward non-endogenous chemical events [17, 180]. Bioorthogonal catalysis can activate therapeutic and imaging agents in inactive counterparts (prodrugs), offering a promising approach for the treatment of diseases [181]. The ability to generate active molecules in situ makes bioorthogonal catalysis ideal for performing localized drug release and therapy, decreasing off-target effects, and improving treatment efficacy [182–184]. Integrating bioorthogonal catalysts into scaffolds produces enzyme-like bioorthogonal nanozymes (BONZs) that can perform diverse non-native transformations under biological environments [17].

**Fig. 14:**
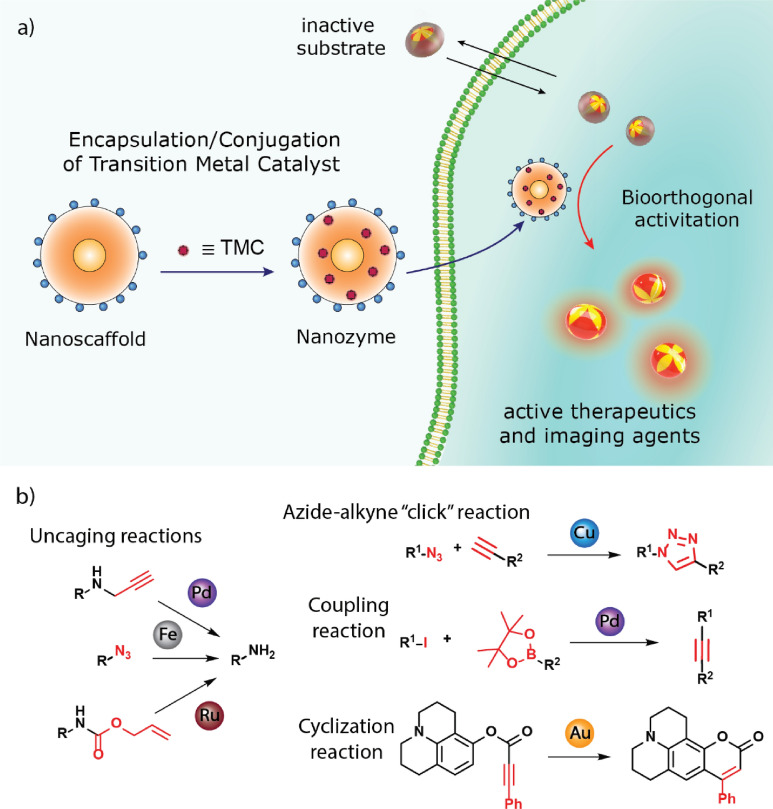
**a** Schematic overview of TMC-based BONZs. After encapsulation and conjugation of the TMC into the nano scaffold, the nanozyme performs the bioorthogonal uncaging of active fluorophores or drug molecules, with nanozyme design dictating localization in vitro and in vivo. **b** Modular nanozyme scaffold hosting different catalysts

Transition metal catalysts (TMCs) provide versatile active sites for BONZs [185, 186]. Usually, TMCs are encapsulated within the nanoparticle scaffolds (Fig. 14a), thereby protecting them from deactivation through enzymatic degradation, extending their lifetime, and increasing the turnover number [187]. Furthermore, the nanoparticle scaffold ensures that the solubility and activity of the TMC in water are maintained by creating a protected microenvironment during the catalysis [188]. Furthermore, the modular nanozyme scaffold can host different catalysts depending on the selected structure, expanding the library of bioorthogonal reactions [180]. Therapeutically inactive substrates can be synthesized by two methods; (1) masking/caging the pharmacophore of a drug molecule with a moiety that is not naturally present in cells and (2) designing inactive substrates that can coupled to generate a therapeutically active drug (Fig. 14b) [186]. Subsequently, the substrate is transformed into its therapeutically active counterpart within the biological environment by the BONZ. The kinetic performance of the reaction often depends on the choice of TMC, substrate, and nano scaffold [180, 189, 190].

In this part, different TMCs, their respective nanozymes, and their application in biomedicine are reviewed. In particular, nanozymes containing Pd-, Au-, Cu-, Ru-, and Fe-based TMCs will be introduced (Table 1).

**Table 1:** TMCs and nano scaffolds used to fabricate nanozymes with their respective bioorthogonal reaction

TMC	Nano scaffold	Bioorthogonal reaction	References
Pd(0), Pd(II)	PLGA-PEG NPs, AuNPs, MoS_2_ nanosheets, ZrO_2_ MOFs	Propargyl uncaging	[191–195]
	Polystyrene NPs, macroporous silica	Suzuki–Miyaura coupling	[196, 197]
	Large pore silica	Transfer Hydrogenation	[183]
Ru	AuNPs, ZnS NPs, Single Chain NPs	Allyl carbamate uncaging	[22, 198, 199]
Cu(I), Cu(II)	Single Chain NPs, CuAl-LDH nanosheets	Azide–alkyne cycloaddition	[200, 201]
	CuS Polydopamine NPs, PdCu NPs	Click reaction, propargyl uncaging	[202, 203]
Fe(III)	Polymeric Nanoparticles (PONI-C_11_-TMA), AuNPs	Azide uncaging reaction	[184, 204, 205]
Au(I), Au(III)	Polymeric Nanoparticles (PONI-C_11_-TMA)	Intramolecular Cyclization	[189]
	Resin NPs	Propargyl Uncaging	[206]

### Pd-based TMCs

Pd-based TMCs can perform well in a wide variety of reactions, including propargyl (alkyne) uncaging, Suzuki–Miyaura cross-coupling, and transfer hydrogenation reactions [187, 207]. Pd-based TMCs can be incorporated into the nanozymes either as homogeneous, like metal organic catalysts and Pd(II), or heterogeneous catalysts, like NPs and Pd(0) [206, 208]. Heterogeneous Pd-based TMCs are highly stable in biological environments [209]. However, Pd NPs may confer in vivo toxicity, particularly in immune, renal, and endocrine systems [210]. In comparison, homogeneous Pd-based TMCs exhibit higher selectivity and faster reaction rates per Pd atom; however, they may interact with DNA molecules, potentially leading to genotoxicity [211, 212].

#### Propargyl uncaging reaction

Prodrugs can easily be produced by masking the pharmacaphore of their corresponding drugs with a protective group [186]. Protective groups, such as propargyl or propargyl carbamate residues, may be attached to amine and alcohol functional groups within the drug molecule [187]. Ideally, the functional group should be involved with or adjacent to the drug pharmacaphore [213]. Prodrugs containing propargyl residues are readily uncaged by Pd(0) and Pd(II) TMCs [186]. Furthermore, the reaction is highly selective due to the absence of propargyl residues in native biological environments [214].

**Fig. 15:**
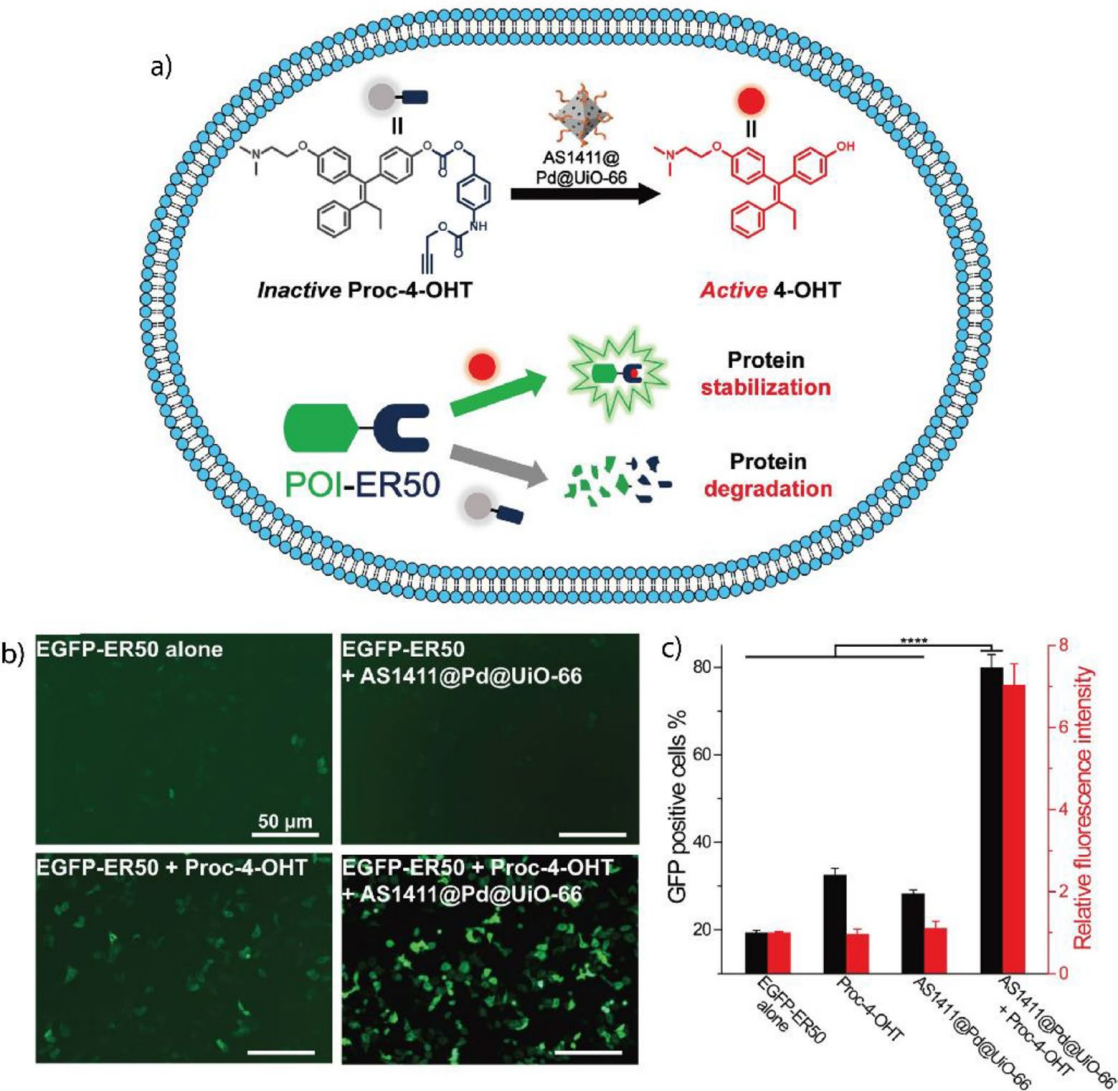
**a** Schematic of the activation mechanism of Proc-4-OHT through the Pd-catalyzed propargyl uncaging reaction. Drug activation stabilized the GFP-labeled protein ER50 in breast cancer cells. **b**, **c** Fluorescence imaging and flow cytometric analysis of ER50-GFP-transfected HeLa cells confirm the protein stabilization upon incubation with the nanozyme and prodrug. Reprinted from Ref. [215] with permission from the American Chemical Society. Copyright 2022

Pd-based TMCs have been encapsulated into various nanomaterials, including polymer, Au, and silica NPs, MOFs, and enzyme cages, to improve their biocompatibility and catalytic performance [191–194, 196, 215–217]. For example, Wang et al. deposited Pd(0) NPs on a zirconium oxide MOF and decorated the surface with a DNA aptamer (AS1411) that targets nucleolin-overexpressing cancer cells (Fig. 15a) [215]. MOFs provide a rigid and stable structure with even lower cytotoxicity than that of other nano scaffolds, such as polymer and silica NPs [218]. Upon interacting with nucleolin receptors on the surface of HeLa cancer cells, the MOFs enter the cell through endosomal uptake. Once inside, MOFs facilitates the uncaging of an anti-cancer drug, propargyl-protected 4-hydroxytamoxifen (Proc-4-OHT), which blocks the use of estrogen by breast cancer cells. 4-OHT regulates the stability of the estrogen-dependent protein ER50 [219]. Green fluorescent protein (GFP)-labeled ER50 was transfected into the HeLa cells. Incubation with the nanozyme and propargyl-protected 4-OHT, led to a ~ ninefold increase in the protein stability, as measured through GFP-fluorescence (Fig. 15b, c).

**Fig. 16:**
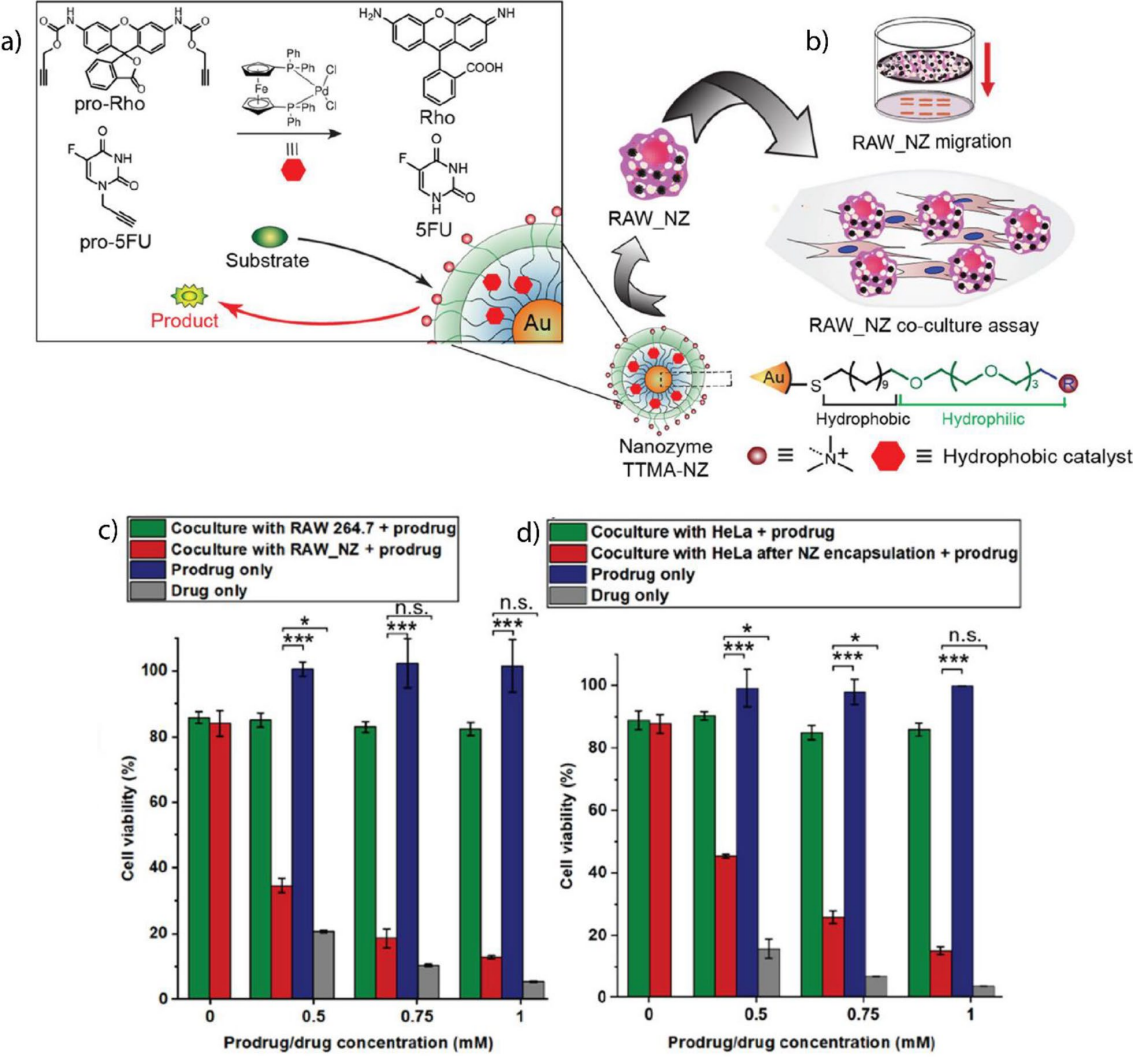
**a** Schematic of the nanozyme-catalyzed activation of a fluorophore Rhodamine 110 and anticancer drug 5-FU and structure of the Au NP ligand. **b** Illustration of nanozyme uptake in macrophages (RAW 254.7) and co-culture assay with HeLa cells. **c**, **d** Comparable efficiency of the HeLa cells and macrophages after the bioorthogonal activation of 5-FU with that of the prodrug approach. Reprinted from Ref. [194] with permission from the American Chemical Society. Copyright 2022

Macrophages are immune cells with different phenotypes and functions. M1-polarized (pro-inflammatory) macrophages inhibit cell proliferation and cause tissue damage for fighting invading species or cells. M2-polarized (anti-inflammatory) macrophages promote cell proliferation and tissue repair [220]. Tumor-associated macrophages (TAMs) are M2-polarized macrophages that protect the tumor from native immune processes and therapeutic interventions [221]. Therefore, targeting TAMs represents a potential therapeutic strategy for tumor treatment. Rotello et al. developed nanozymes based on Au NPs decorated with an organic ligand consisting of hydrophobic and hydrophilic segments, and a cationic headgroup (Fig. 16a, b) [194]. The hydrophobic segment facilitates the encapsulation of the homogeneous Pd-based TMCs, the hydrophilic segment increases the solubility of the nanozyme in water, and the cationic headgroup enables the cellular uptake of the nanozyme. The nanozymes were taken up by the TAMs and facilitated the release of a propargyl-protected 5-fluorouracil (5-FU) derivative in a co-culture model with HeLa cells, showing a comparable efficiency to that of the free drug (Fig. 16c, d). The study proposed an innovative approach using nanozymes carried by TAMs for improved localized cancer therapy, potentially decreasing drug-mediated off-target effects.

**Fig. 17:**
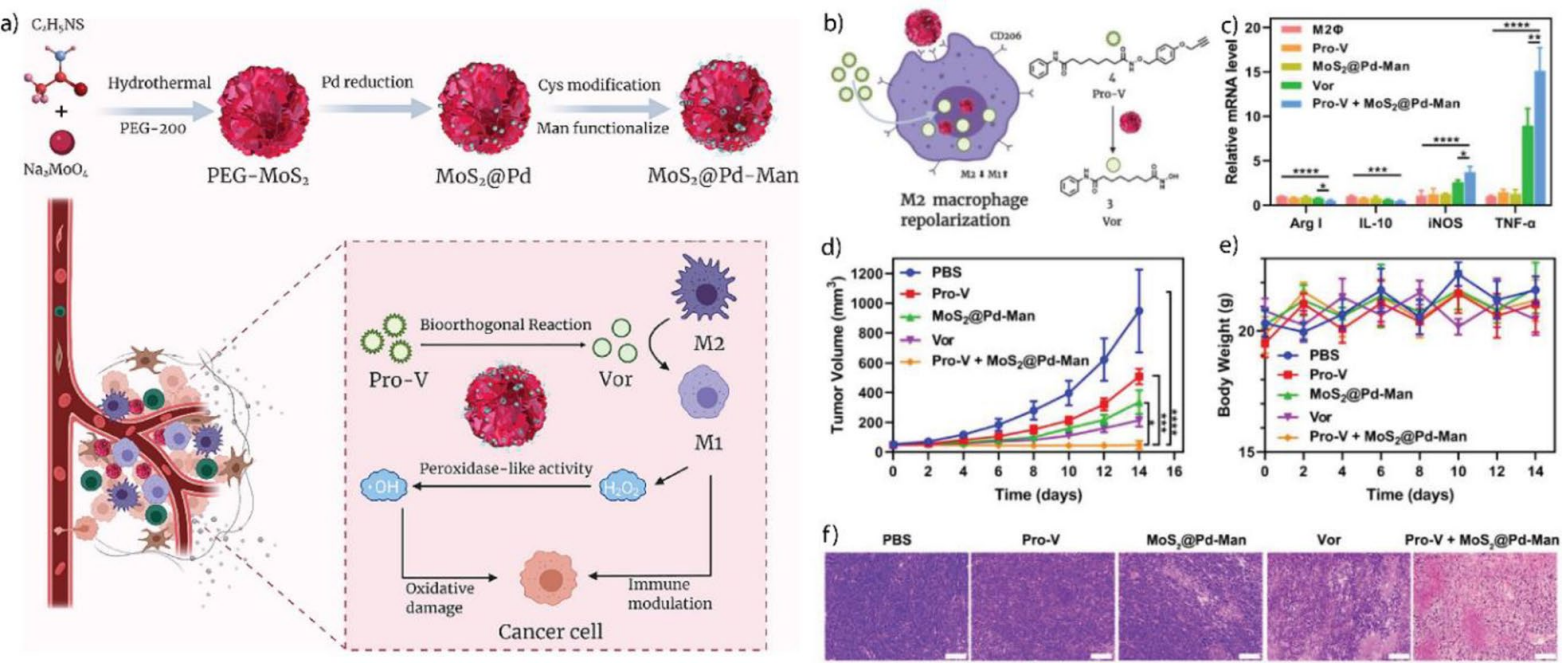
**a** Schematic of the preparation of the nanozyme and bioorthogonal release of Vorinostat in tumor to initiate macrophage repolarization. **b** Mannose-functionalized nanozymes are uptaken by cancer cells and mediate the uncaging of propargyl-protected Vorinostat (Pro-V). **c** Similar levels of Arg I and IL-10 and considerably increased levels of iNOS and TNF-α in the phenotype markers of the treated macrophages compared to that of the free drug. **d** Improvement of treatment efficiency based on tumor volume after treatment with the nanozyme and prodrug relative to those of the free drug and controls. **e** Body weight of mice during treatment. **f** Staining of tumors with hematoxylin and eosin (H&E) to observe the changes in tumor tissue after treatment. Reprinted from Ref. [195] with permission from Elsevier Inc. Copyright 2022

Small molecules, such as the FDA-approved anticancer drug Vorinostat, can modulate the polarization of macrophages and change their behavior and function from an M2 to an M1 type [195]. Qu et al. deposited Pd(0) NPs on MoS_2_ NPs with a water-soluble polyethylene glycol ligand (Fig. 17a). The Pd-based TMC catalyzed the uncaging of a propargyl-protected Vorinostat derivative, efficiently repolarizing M2-type macrophages (Fig. 17b, c). In vivo testing using a colon-cancer model (CT26 cells) showed that the nanozymes prevented tumor growth for up to 14 days of treatment (Fig. 17d–f).

#### Suzuki–Miyaura cross-coupling reaction

Suzuki–Miyaura cross-coupling is the reaction between boronic acids or esters and alkene, arene, or alkyne halides [17]. This coupling reaction is useful for the bioorthogonal formation of drug molecules [187]. The assembly of drug molecules from multiple individual parts can provide a significant therapeutic window due to the general lack of activity of the respective molecular parts [222]. The cross-coupling reaction can progress orthogonally to other Pd-catalyzed reactions [197].

**Fig. 18:**
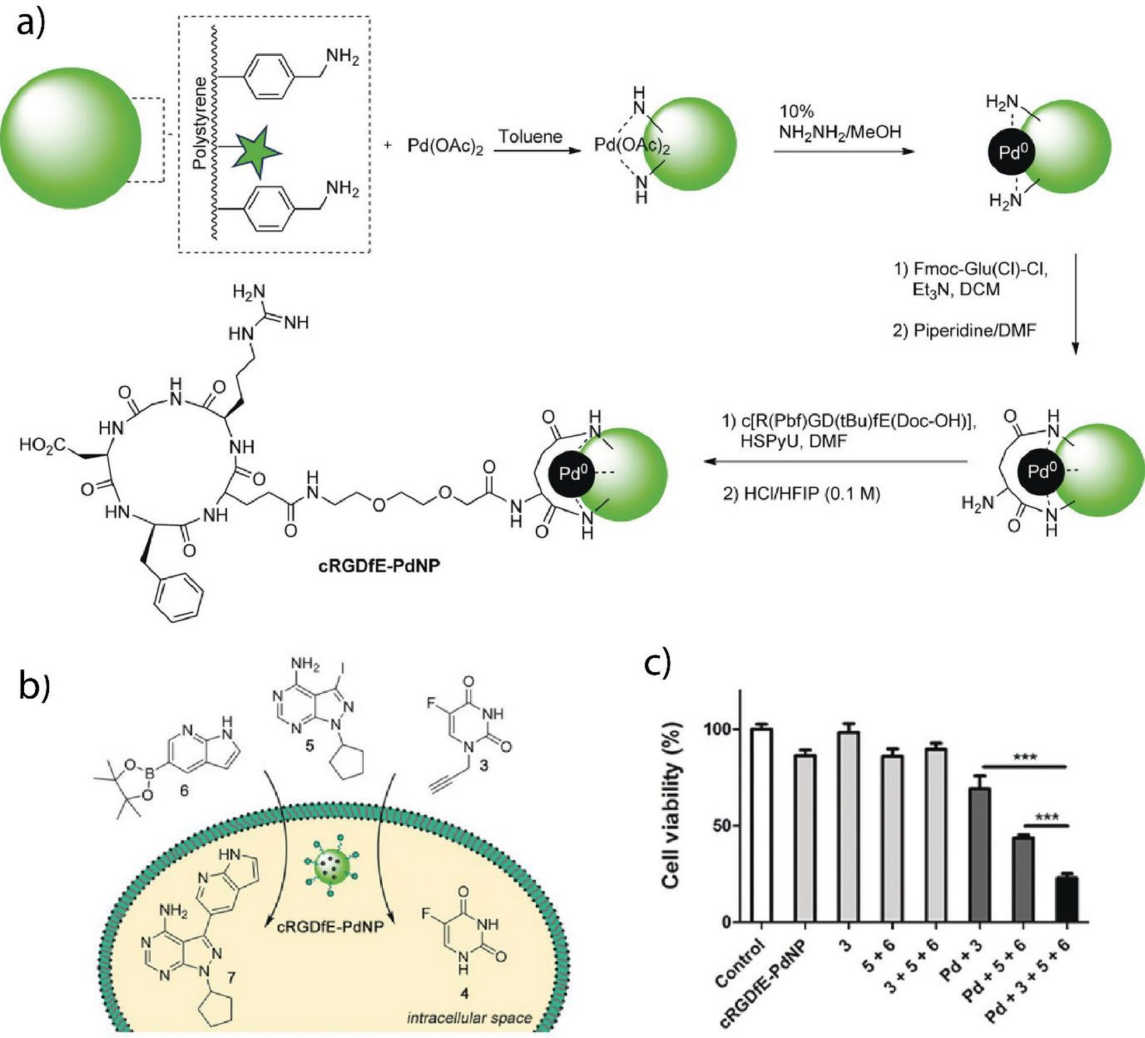
**a** Schematic the preparation process for the Pd-based nanozyme. **b** Uncaging of propargyl-protected 5-FU using the nanozyme. Simultaneous drug release of anticancer agents PP-121 and 5-FU mediated by nanozyme. **c** Cell viability of U87-MG cells after treatment with nanozyme and prodrugs separately and concurrently. Reprinted from Ref. [197] with permission from Wiley–VCH. Copyright 2017

Bradley et al. prepared polystyrene NPs with surface primary amine groups that coordinated with Pd(0) NPs and decorated the exterior with a cyclic peptide, cRGD, which served as a targeting ligand for α_v_β_3_ receptor-overexpressing tumors (Fig. 18a) [197]. The nanozyme facilitated the uncaging of propargyl-protected 5-FU and concurrently assembled the anticancer agent PP-121 through the Suzuki–Miyaura cross-coupling reaction. It was used to perform combination drug release in human glioblastoma (U87-MG) cells (Fig. 18b). The combination treatment exhibited synergistic effects compared to individual treatments using 5-FU and PP-121 (Fig. 18c).

**Fig. 19:**
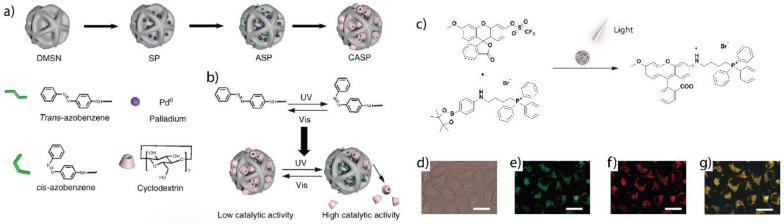
**a** Schematic of the synthesis process of the nanozyme using macroporous silica nanoparticles. **b** UV-induced uncapping of the nanozymes due to the change in azobenzene conformation. **c** Bioorthogonal synthesis of mitochondria-targeting Rhodamine 110 derivative after the UV-induced activation of nanozymes. **d** Bright-field image of HeLa cells. **e** Nanozyme-catalyzed release of the fluorophore. **f** Cells incubated with MitoTracker® Ref CMXRos. **g** Merging of (**e**) and (**f**) to show the colocalization of the fluorophore and the mitochondrion. Reprinted from Ref. [196] with permission from Springer. Copyright 2018

Pd-based TMCs exhibit a versatile coordination chemistry that can be used to coordinate functional groups and pre-organize catalytic sites [196]. Qu et al. deposited Pd(0) TMC on macroporous silica nanoparticles (DMSN), coordinated azobenzene arms to the TMC, and finally capped the catalyst with cyclodextrin to reduce their activity them by making them inaccessible to the substrate (Fig. 19a). Under UV irradiation, the conformation of the azobenzene arms changed from *trans*- to *cis*-, releasing the cyclodextrin caps and restoring the inherent catalytic activity. When the UV irradiation was stopped, the conformation of the azobenzene arms reverted to *trans*-, allowing the re-coordination of the cyclodextrin caps (Fig. 19b). This ON/OFF-switchable nanozyme demonstrated potential in HeLa cells by releasing a mitochondria-specific fluorophore through the Suzuki–Miyaura cross-coupling reaction (Fig. 19c–g).

#### Transfer hydrogenation reactions

Pd-based TMCs can use hydrogen sources, such as nicotinamide adenine dinucleotide-hydrogen (NADH) and HCOONa, to perform transfer hydrogenation reactions for the reduction of double bonds [183]. When chiral modifiers participate in the catalysis, the reaction can be performed with high enantioselectivity, producing an excess of one enantiomer [223]. This strategy can be of use in the synthesis of chiral drug molecules, including non-steroidal anti-inflammatory drugs like ibuprofen and ketoprofen [224]. Additionally, nanozymes can be decorated with targeting moieties that may accumulate at an inflammation site, increasing the efficiency of localized treatment [225].

**Fig. 20:**
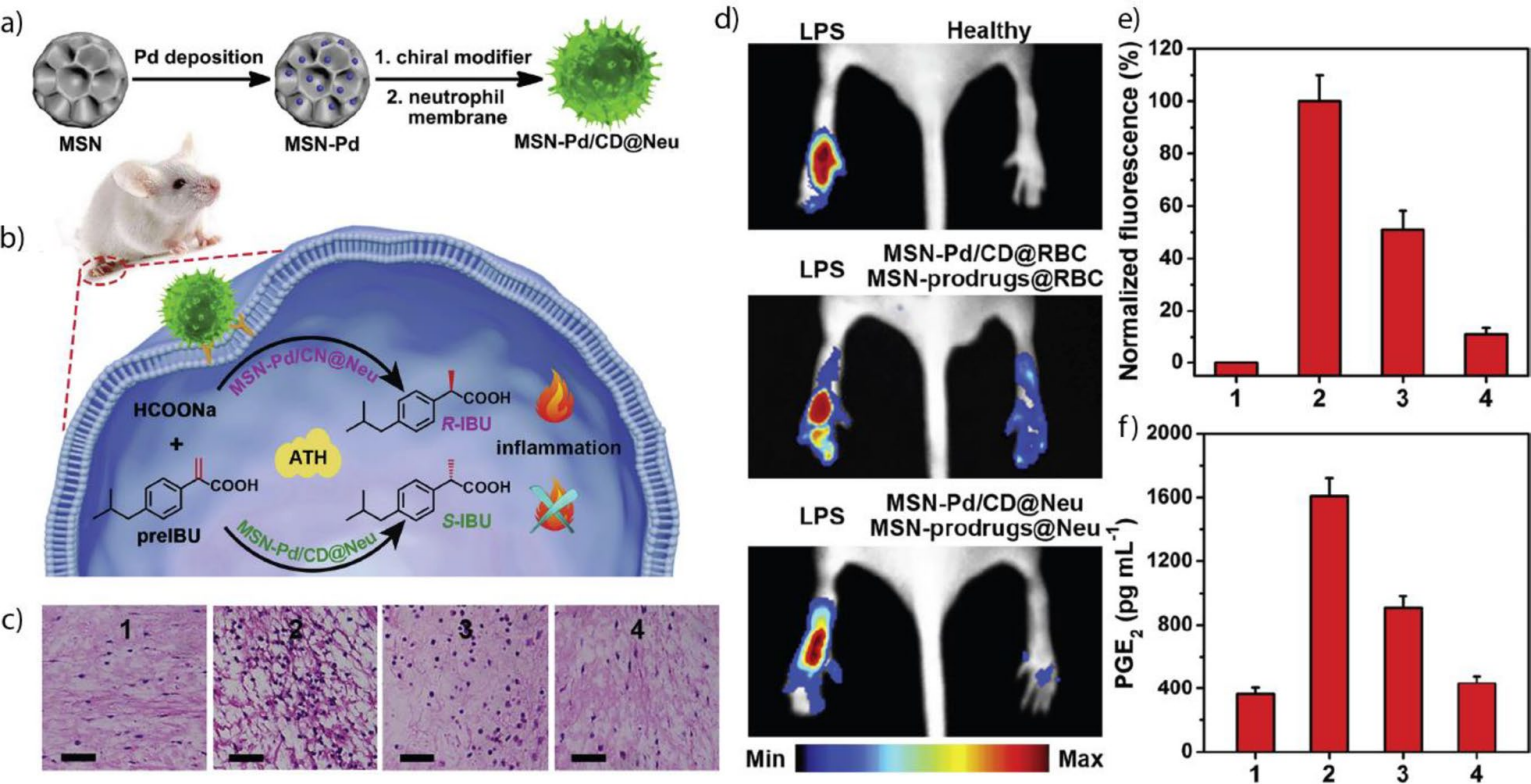
**a** Schematic of the nanozyme fabrication process. **b** Intracellular activation of ibuprofen mediated by the chiral nanozymes. **c** H&E staining of mouse paws treated with the (1) saline, (2) LPS, (3) LPS and the CD-nanozyme, and (4) LPS, CD-nanozyme, and prodrug. Inflammation-inducing conditions caused extensive morphological damage of the tissue. CD-nanozyme and prodrug reduced inflammation and preserved the morphology of the healthy tissue. **d**) ROS imaging of untreated, treated, and control mouse paws. **e** ROS reduction quantified through fluorescence measurement of the ROS-responsive dye, DCFH-DA. **f** PGE_2_ levels of the respective treatment groups and controls. Reprinted from Ref. [183] with permission from Elsevier Inc. Copyright 2022

Qu et al. fabricated nanozymes based on large-pore silica NPs with deposited Pd(0) NPs (Fig. 20a) [183]. The surface was functionalized with the chiral modifiers, cinchonidine (CD) and cinchonine (CN) to produce two enantioselective nanozymes. Furthermore, the nanozyme surfaces were coated with neutrophil cells derived from mice to direct the accumulation at an inflammation site. Lipopolysaccharide (LPS) was introduced to BALB/c mouse paws to mimic inflammatory conditions. Then, the inflammation was treated locally using the nanozyme and ibuprofen precursor. The respective nanozymes facilitated the asymmetric transfer hydrogenation of the ibuprofen precursor, releasing either the active *S*- or inactive *R*-ibuprofen (Fig. 20b). Using CD-modified nanozymes released *S*-ibuprofen, efficiently decreasing prostaglandin E_2_ (PGE_2_) and ROS concentrations and mitigating inflammation (Fig. 20c–f).

### Ru-based TMCs

Ru-based TMCs have high stability and longevity in biological media and can perform allyl and allyl carbamate uncaging reactions, azide–thioalkyne cycloadditions, and olefin metathesis in aqueous environments [226, 227]. Furthermore, Ru-based TMCs can be designed to undergo light-induced activation, allowing the fabrication of photo-responsive nanozymes [227]. However, compared to Pd TMCs, Ru TMCs possess significantly higher toxicity, which limits their use as bioorthogonal TMCs [228]. Additionally, free Ru TMCs are vulnerable to endogenous thiols and may deactivate due to ligand exchange [187].

**Fig. 21:**
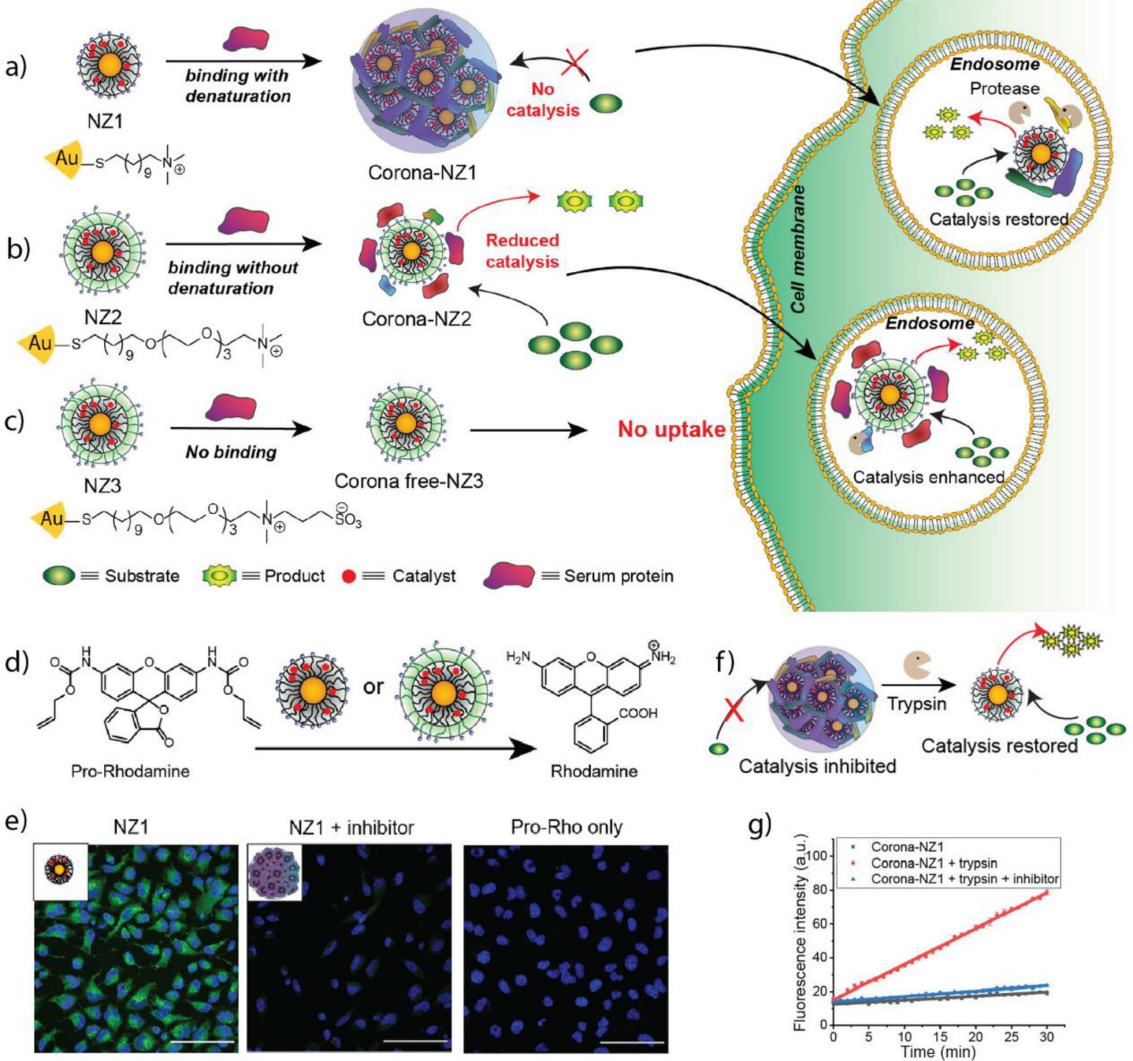
**a** Schematic of the mechanism of hard corona formation and catalysis inhibition before intra-endosomal reactivation. **b** Soft corona formation and enhancement of catalysis upon endosomal uptake. **c** Inhibition of endosomal uptake due to the presence of a zwitterionic headgroup on the nanozyme. **d** Nanozyme-mediated Rhodamine 110 uncaging. **e** Confocal imaging of the HeLa cells after 8 h of incubation with the nanozyme and pro-Rho, and a control group with a protease inhibitor. Endosomal proteolysis is crucial for restoring catalytic activity. **f** Schematic of the trypsin-mediated reactivation of nanozyme catalysis. **g** Quantification of the dye release of the hard corona nanozyme, the nanozyme after proteolysis, and the control with a protease inhibitor through fluorescence spectroscopy. Reprinted from Ref. [22] with permission from the American Chemical Society. Copyright 2020

Encapsulating Ru-based TMCs into nano scaffolds can protect the metal center from free thiols, prolonging the lifetime of the catalyst [229]. Furthermore, designing an outer shell may form a protective corona around the nanozyme in serum, shielding the access of the substrate to the catalyst and thereby blocking catalysis [22]. Rotello et al. decorated Au NPs with hydrophobic ligands to form a protein corona around the nanozyme in serum (Fig. 21a–c). Depending on the structure of the ligand used, the formation of a hard (irreversible) or soft (reversible) protein corona was observed. After the encapsulation of a Ru-based TMC ([CpRu(8HQ)(allyl)PF_6_), the nanozymes effectively catalyzed the uncaging of allyl carbamate-protected Rhodamine 110 (Fig. 21d). However, catalysis was quenched due to the presence of the hard protein corona in the serum. After the endosomal uptake, the protein corona was degraded by proteases, leading to the reactivation of catalysis (Fig. 21f, g). The nanozyme successfully activated Rhodamine 110 in HeLa cells (Fig. 21e).

**Fig. 22:**
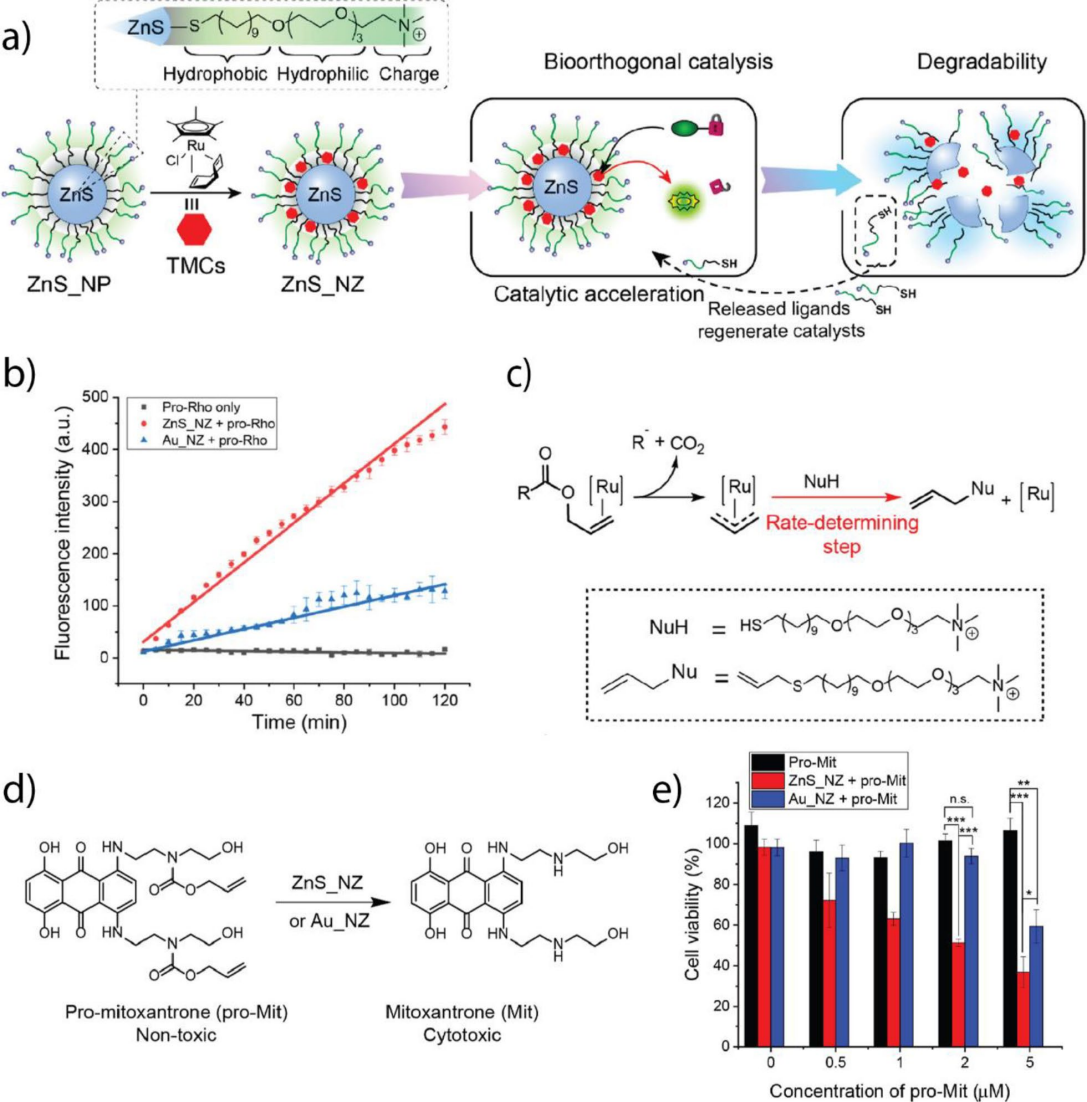
**a** Nanozyme structure and schematic of the encapsulation of the Ru-based TMC. After nanoparticle degradation, the release of ligands increases the catalytic rate due to the presence of nucleophiles. **b** Comparison between the catalytic performance of Au and ZnS nanozymes. **c** Rate-determining step of catalysis involves the conjugation of a nucleophile to the cleaved residue. **d** Uncaging reaction of the propargyl-protected anticancer drug mitoxantrone. **e** Higher cell killing efficiency of ZnS nanozyme than that of Au nanozyme after the prodrug activation. Reprinted from Ref. [198] with permission from the American Chemical Society. Copyright 2022

Rotello et al. also developed and designed biodegradable nanozymes based on ZnS NPs [198]. The particle surface was decorated with an organic ligand consisting of a hydrophobic segment, a hydrophilic segment, and a cationic headgroup (Fig. 22a). Upon degradation of the ZnS NPs on the surface, the thiol-containing ligand is released and acted as an additional nucleophile, accelerating the catalysis. Similarly, the Ru-based TMC ([CpRu(8HQ)(allyl)PF_6_) was encapsulated with the biodegradable enzyme. Thereafter, the activities of the ZnS- and Au NP-based nanozymes were compared. The biodegradable nanozyme exhibited 250 times higher activity than that of the Au NP-based nanozyme toward the catalysis of the uncaging of the allyl carbamate-protected Rhodamine 110 (Fig. 22b, c). The catalytic activity of the ZnS-based nanozyme for the catalytic activation of the allyl carbamate-protected anticancer drug Mitoxantrone in HeLa cells was also tested. The cell-killing efficiency of the ZnS nanozyme was significantly higher cell killing than that of the Au nanozyme, indicating the effectiveness of this approach and addressing the safety issues related to the slow biodegradation of nanozyme scaffolds such as Au (Fig. 22d, e).

Furthermore, BONZs with responsiveness to external stimuli were designed by Rotello et al. to produce bioactive agents in a bioorthogonal manner upon encountering specific exogenous triggers [230]. For instance, BONZ containing ligands featuring a quaternary ammonium group were rendered inactive through host–guest cucurbituril-complexation. The complexation of the headgroup obstructed the access of the substrate to the Ru-based TMC within the AuNP monolayer, leading to the inhibition of catalysis. Introducing adamantylamine that binds strongly to the cucurbituril competitively removed the blocking group, restoring BONZ'catalytic activity. This adamantylamine-triggered catalytic activation was successfully demonstrated within cells, using a pro-dye to monitor the activity, and the therapeutic potential was showcased through the activation of an anticancer prodrug (pro-5FU) specifically within tumor cells.

**Fig. 23:**
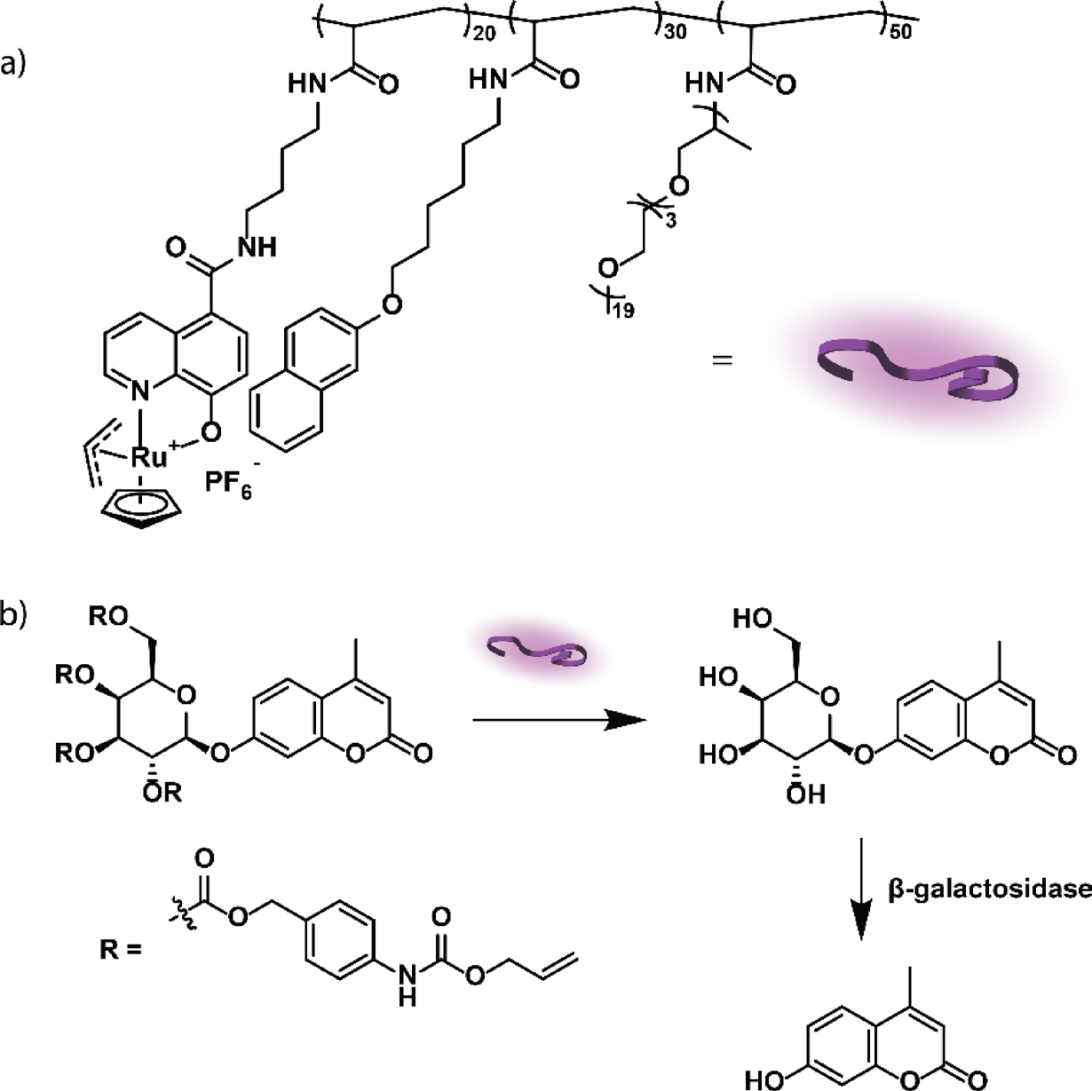
**a** Structure of SCNP and its functionalization with quinoline, naphthalene, and Jeffamine M-1000, followed by the coordination of the Ru-based TMC. **b** Tandem-catalysis mediated by the Ru-based TMC (1^st^ step) and β-galactosidase (2^nd^ step) for the activation of 7-hydroxy coumarin. Redrawn from Ref. [199]

Ru-mediated allyl carbamate uncaging reactions follow a distinct catalytic mechanism that can occur concurrently with other reactions, such as hydrolysis and cyclization [231]. Zimmerman et al. synthesized a single-chain nanoparticle (SCNP) with a covalently bound Ru-based TMC, [CpRu(MeCN)_3_]PF_6_ [199]. The scaffold was prepared using an amphiphilic copolymer based on a poly(pentafluoro phenyl acrylate). The catalyst was attached via an amide bond (Fig. 23a). A non-fluorescent coumarin derivative was synthesized by protecting 7-hydroxy coumarin with 4-methylumbelliferyl-β-D-galactopyranoside and then, by introducing allyl carbamate phenyl carbonate groups to hydroxy-groups on the sugar (Fig. 23b). Catalysis using SCNP was performed in the presence of β-galactosidase, leading to the Ru-mediated uncaging of the allyl carbamate phenyl carbonate residues and hydrolysis of the sugar, and restoring fluorescence. The tandem catalysis achieved more than 60% catalytic conversion within 20 min under physiological pH conditions.

### Cu-based TMCs

Cu(I)-based TMCs can facilitate bond formation between aryl and azide residues that are not native to biological environments [187]. The pioneering work of Bertozzi et al*.* in bioorthogonal chemistry involved the by development and application of azide–alkyne "click" reactions to label proteins, DNA, and sugars, greatly impacting biology and medicine [232].

**Fig. 24:**
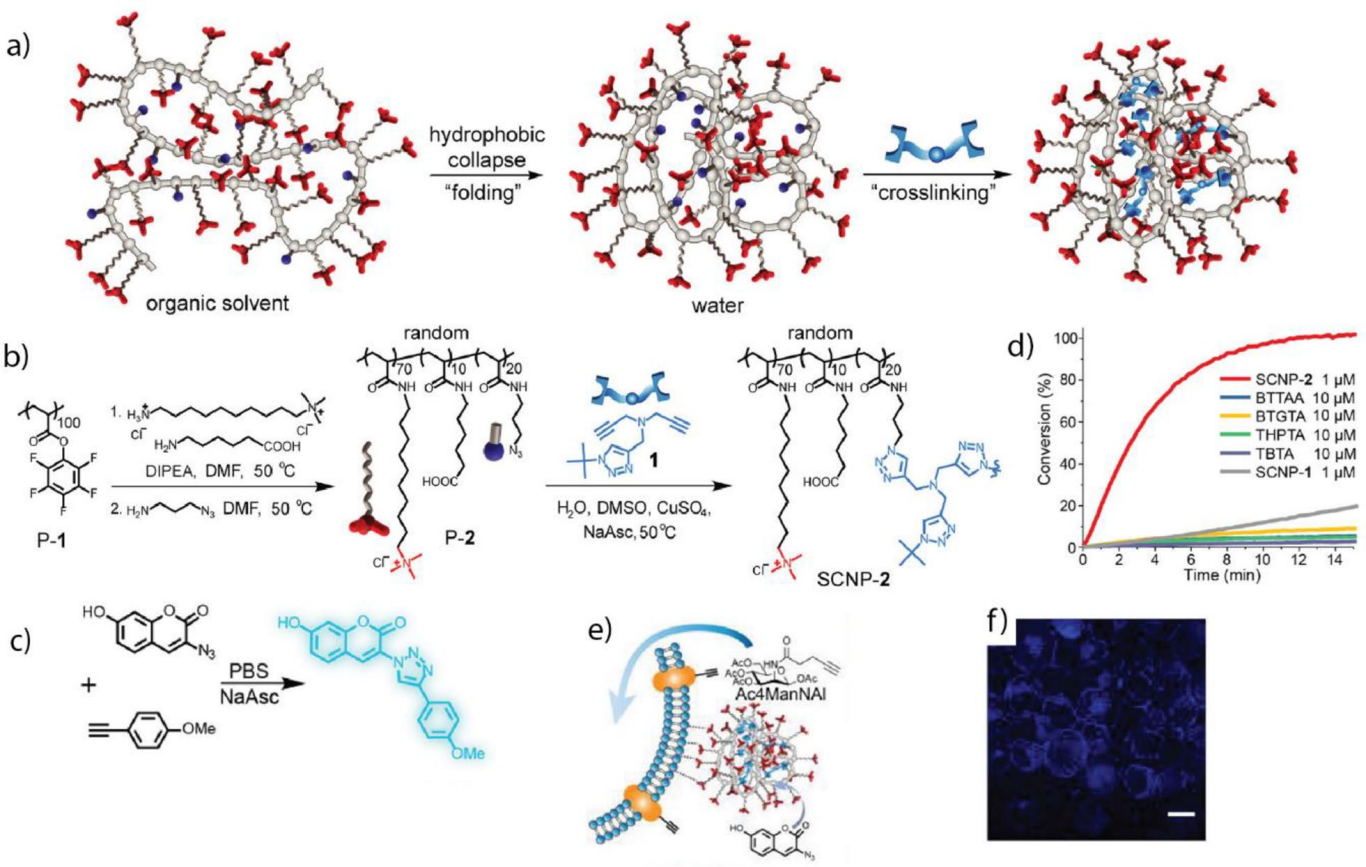
**a** Schematic of the synthesis process of the Cu SCNP. **b** Schematic of the SCNP functionalization and cross-linking with a Cu-chelating ligand. **c** SCNP-mediated "click" reaction that formed the fluorescent coumarin derivative. **d** Catalytic performance of SCNPs with different cross-linking moieties. **e** Schematic of the "click" reaction mechanism between propargyl-functionalized glycans in the cell surface and azide-functionalized coumarin. **f** Confocal images of the cell surface labeled with H460 cells after incubation with Cu-SCNP, azide-functionalized coumarin, and sodium ascorbate. Reprinted from Ref. [200] with permission from the American Chemical Society. Copyright 2019

Bioorthogonal "click" reactions can proceed without a catalyst but their rates can be considerably improved when a Cu-based TMC is used [233]. However, free Cu(I) can induce cyto- and genotoxicity by catalyzing the formation of ROS that damage biomolecules [234]. As such, encapsulating Cu(I) into a nano scaffold may reduce the toxic potential while maintaining the catalytic activity of the metal. Zimmerman et al. fabricate SCNPs based on Cu(I) coordinated to a chelating ligand, which was covalently bound to a polymeric nano scaffold [200]. The positively charged trimethylamine group was introduced into the SCNP to improve its interaction with biological membranes (Fig. 24a–e). The incubation of H460 cells with mannose molecules containing aryl-residues led to the metabolic implementation of the mannose into the surface glycans of the cells. As confirmed through confocal microscopy, SCNPs selectively labeled these surface glycans by performing the "click" reaction between the present aryl-residues and an azide-containing coumarin (Fig. 24f).

**Fig. 25:**
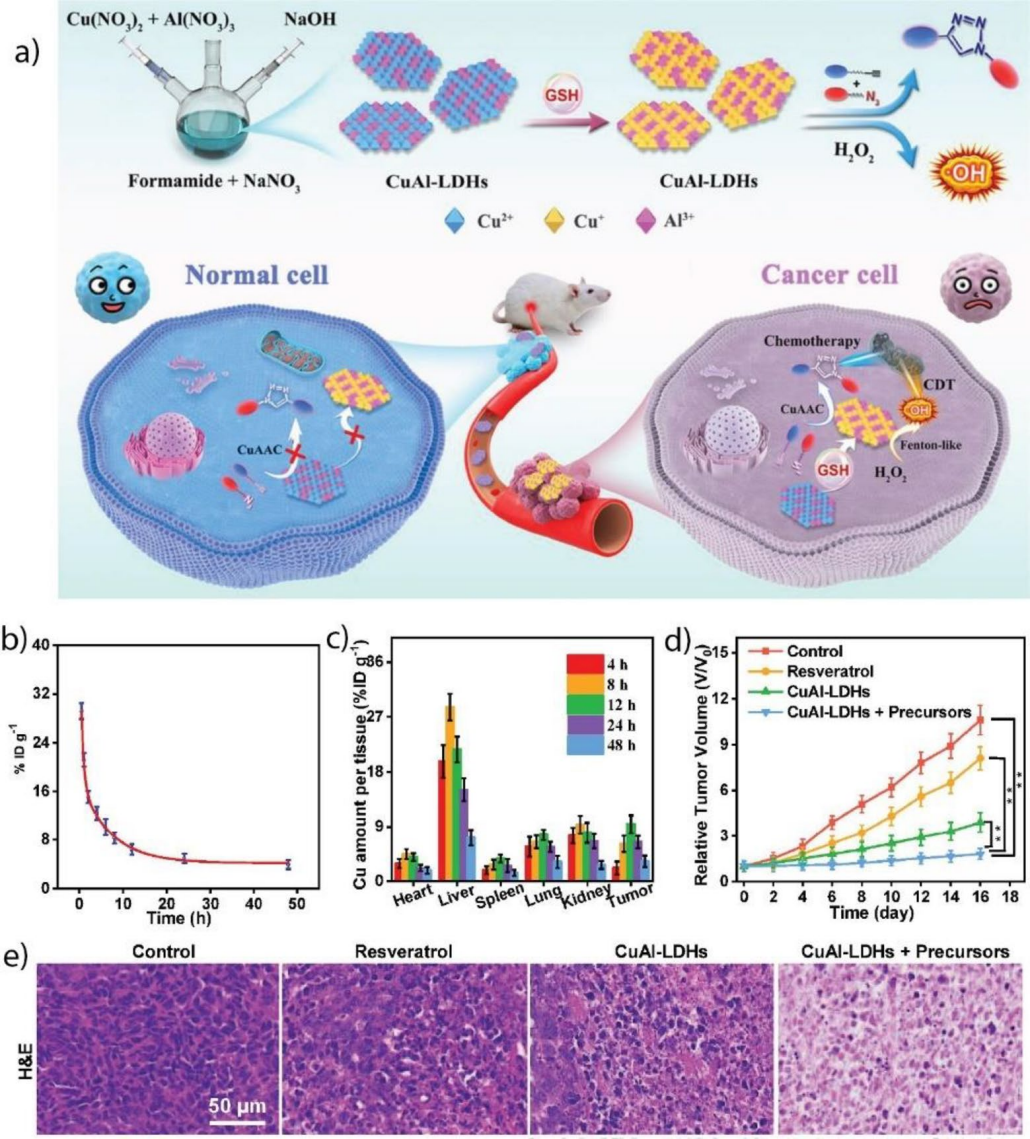
**a** Schematic of the formation of the CuAl-LDH and GSH-induced enhancement of catalysis in cancer cells. **b** Pharmacokinetic curve of nanosheets, showing the concentration of the nanozyme in the blood plasma as a function of time. The blood circulation halftime of the nanosheets was approximately 7.23 ± 0.24 h. **c** Biodistribution of CuAl-LDH, showing its high concentrations in tumors, lungs, liver, and kidneys. **d** Tumor volume in 4T1 tumor-bearing mice (control groups) and activation group treated with the resveratrol precursors and nanozyme. **e** H&E staining showing the difference in tissue morphology in the control and treatment group and indicating substantially less tissue damage in the nanozyme activation group than in the controls. Reprinted from Ref. [201] with permission from Elsevier Inc. Copyright 2022

Stimuli-responsive activation can also be performed to reduce the toxicity of Cu in Cu-based TMCs. Cu-based TMCs have their redox-catalytic potential in biological environments that contain large amounts of reducing agents, such as glutathione and cysteine [201]. Tumor microenvironments contain high levels of endogenous thiols due to increased ROS production from the rapid proliferation and increased metabolism of cancer cells [235]. Recently, Chen et al. developed CuAl-layered double hydroxide (CuAl-LDH) nanosheets that responded to intracellular thiols as endogenous stimuli [201]. The Cu-based TMC embedded in the nanosheets is not catalytically active owing to the presence of Cu(II). Catalysis was activated when the endogenous thiols reduced Cu(II) to Cu(I). The CuAl-LDH nanosheets acted as bioorthogonal and biomimetic nanozymes that synergistically catalyzed the formation of a resveratrol analog, increasing the intracellular concentration of H_2_O_2_ and subsequently releasing ROS through a Fenton-type reaction (Fig. 25a). The intratumorally implanted-nanosheets reduced tumor growth and improved survival rates in 4T1 tumor-bearing mice (Fig. 25b–e), demonstrating the potential of therapeutic approaches that combine bioorthogonal and biomimetic strategies.

**Fig. 26:**
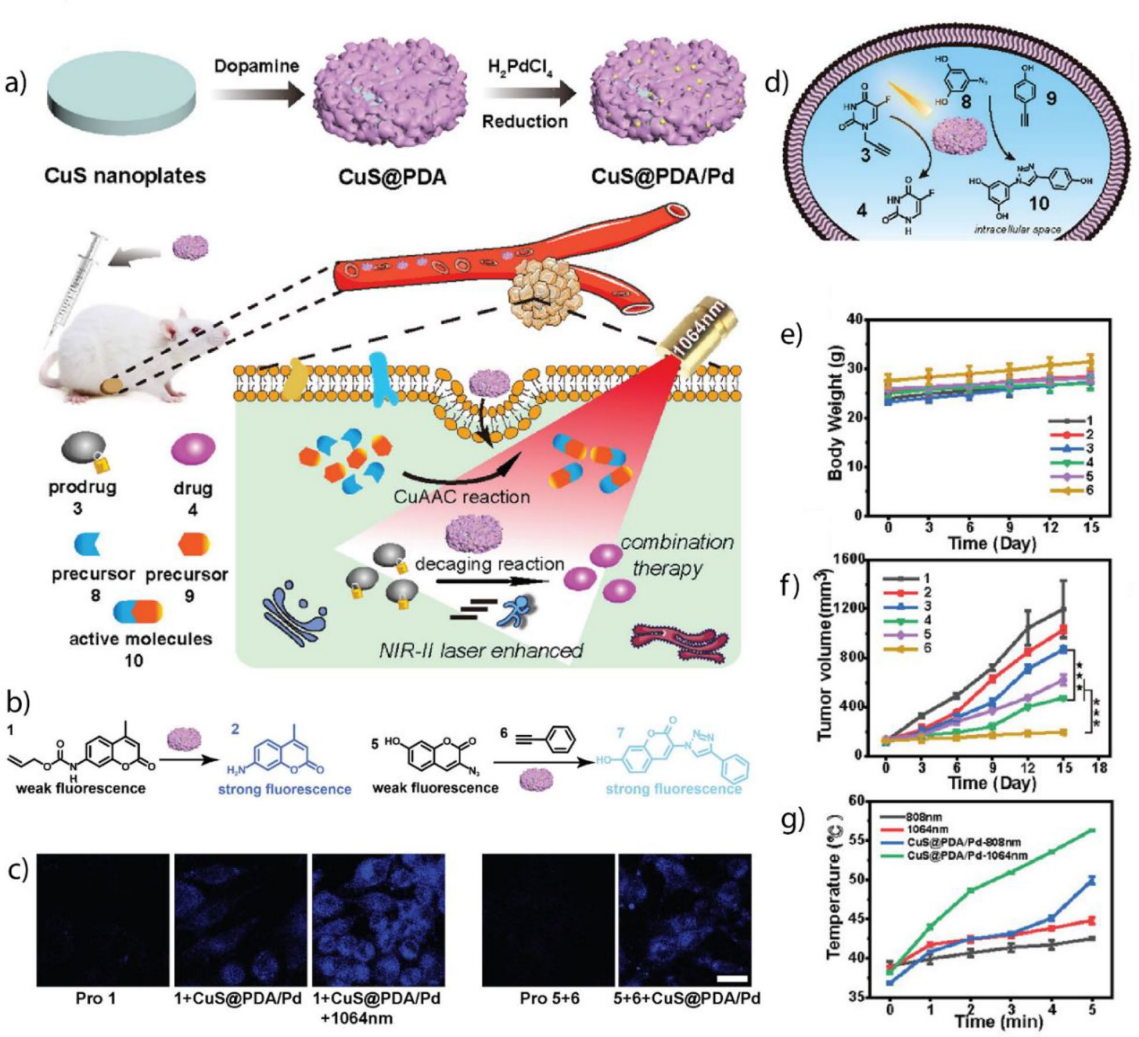
**a** Schematic of the nanozyme fabrication and enhancement of combination drug release using a near IR laser. **b** Pd-catalyzed propargyl uncaging of a coumarin derivative and Cu-mediated azide–alkyne "click" reaction of a coumarin triazole species. **c** Independent confocal imaging of both fluorophores, demonstrating the enhanced Pd activity upon near IR irradiation. **d** Concurrent intracellular release of anticancer drugs. **e** Body weight of 4T1 tumor-bearing mice in different treatment groups: (1) PSB control and 808 nm light, (2) nanozyme and 808 nm light, (3) PBS control and 1064 nm light, (4) nanozyme and 1064 nm light, (5) nanozyme, prodrugs, and 808 nm light, (6) nanozyme, prodrugs, and 1064 nm light. **f** Changes in tumor volume for the same treatment groups as described in e), demonstrating the considerably decreased tumor growth upon treatment with nanozyme, prodrugs, and 1064 nm light. **g** Temperature changes in the tumors of 4T1 tumor-bearing mice as a function of the irradiation time. Reprinted from Ref. [202] with permission from the American Chemical Society. Copyright 2022

Exogenous stimuli, such as ultrasound, heat, or light, can also induce stimuli-responsive bioorthogonal catalysis [202]. Depending on the laser intensity, near IR light can penetrate up to 5 mm deep into human tissue without damaging the tissue itself, making it an attractive exogenous stimulus for anticancer therapy [236]. Qu et al. developed a near IR responsive-nanozyme that can simultaneously catalyze azide-aryl "click" and propargyl uncaging reactions [202]. CuS and Pd NPs were loaded in polydopamine and polymer scaffolds, respectively (Fig. 26a–c). The CuS NPs catalyze the assembly of resveratrol, while the Pd NPs facilitate the uncaging of propargyl-protected 5-FU. Near IR light accelerates catalysis due to the photothermal heating of the NPs. Simultaneous drug release in 4T1 tumor-bearing mice considerably decreased tumor growth rates for up to 15 days of treatment (Fig. 26d–g).

**Fig. 27:**
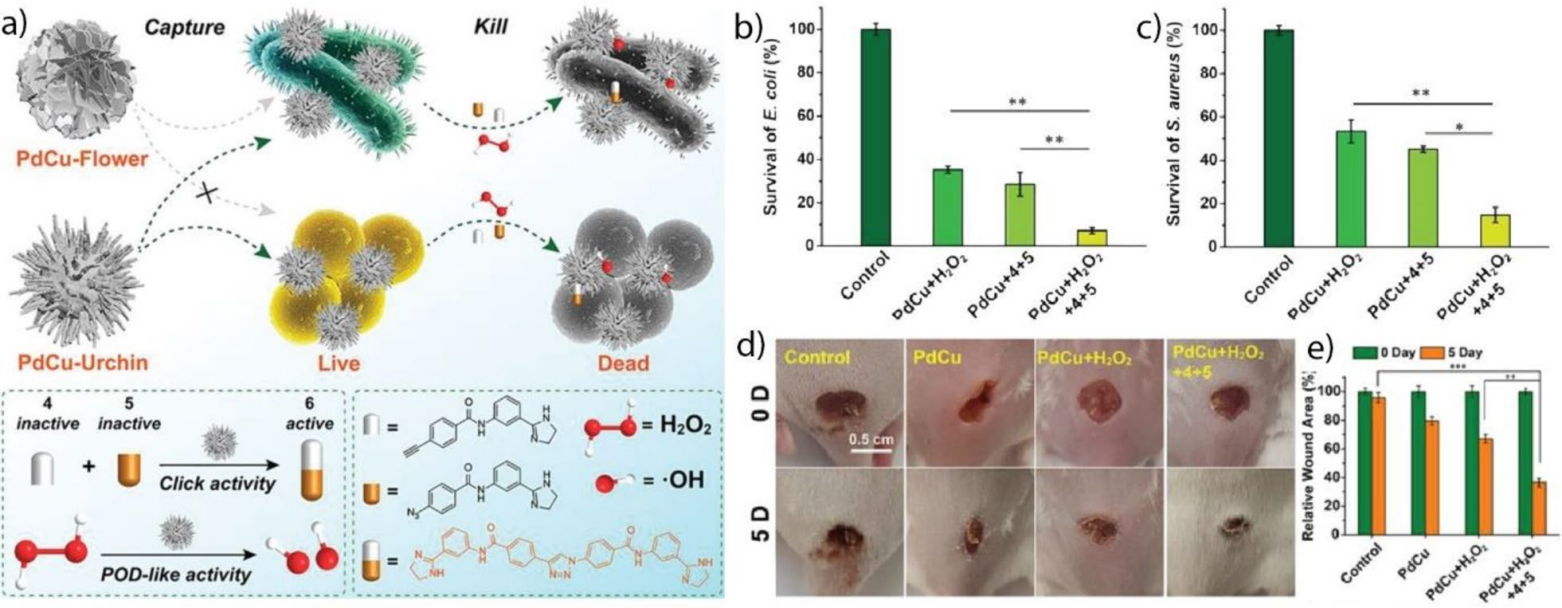
**a** Schematic of the bacteria capture and drug activation through via azide–alkyne "click" reaction and ROS release using the sea urchin-like PdCu nanozymes with POD-like activity. **b**, **c** Survival rate of *E. coli* and *S. aureus* after treatment with the nanozyme and H_2_O_2_ or drug precursors, compared to combination treatment. **d** Photographs of *E. coli*-infected mouse wound models under control and combination treatment. **e** Relative wound area at Day 0 and Day 5 of treatment. Reprinted from Ref. [203] with permission from the American Chemical Society. Copyright 2021

Pd- and Cu-based TMCs can be used in combination in a synergistic therapeutic strategy [203]. Using antimicrobial drugs for the treatment of bacterial infection may form drug-resistant bacteria, reducing the therapeutic efficiency [237]. Producing ROS using metal NPs can kill bacteria non-specifically and without the formation of drug resistance [238]. However, non-specific ROS treatment damages surrounding tissue, limiting the feasible ROS release in humans. Qu et al. developed urchin-like PdCu NPs that can catalyze the bioorthogonal release of an antimicrobial agent and promote the local production of ROS in the presence of H_2_O_2_ (Fig. 27a) [203]. Urchin-like PdCu NPs showed a synergistic effect in treating *E. coli* wound biofilm in vivo, compared to the separate treatments (Fig. 27b–e).

### Fe-based TMCs

Fe-based TMCs possess low toxicity compared to heavy metals such as Pd(II), Au(III), and Cu(I) because of their natural presence in vivo, such as in the heme group of hemoglobin [239]. Fe-containing organometal complexes can act as redox catalysts, reducing azide- or nitro-residues to their respective primary amine [240, 241]. Fe-mediated redox catalysis has a high turnover rate, allowing the rapid release of substrate molecules [242].

**Fig. 28:**
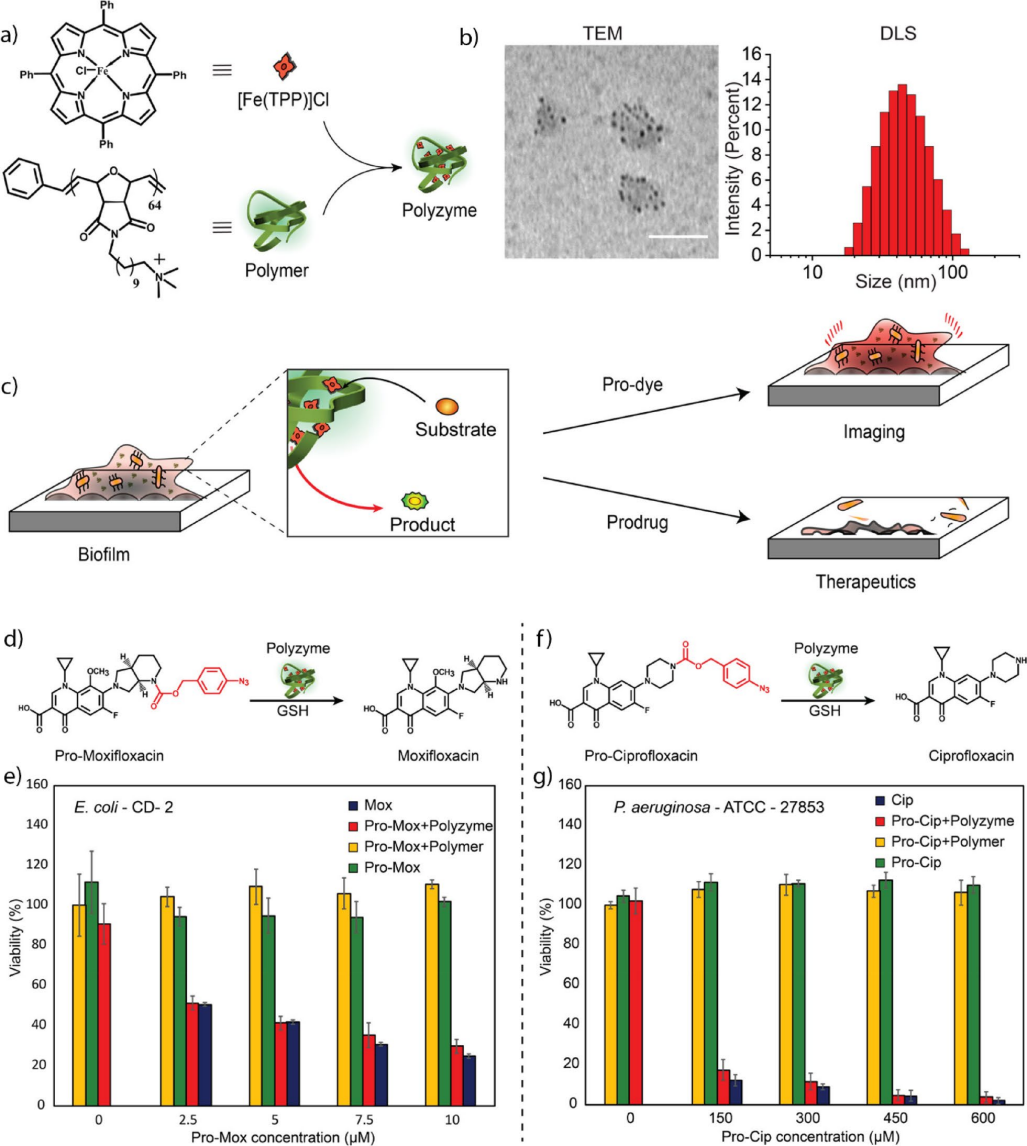
**a** Nanozyme fabrication using a PONI-C11-TMA and FeTPP. **b** Transition electron microscopy and dynamic light scattering (DLS) characterization of nanozymes. **c** Schematic of the fluorophore and antimicrobial drug release in the bacterial biofilm mediated by the nanozyme. **d**, **e** Bioorthogonal release of moxifloxacin and bacteria viability of *E. coli* biofilm after treatment with prodrug and nanozyme demonstrates comparable efficiency to that of the free drug. **f**, **g** Bioorthogonal release of ciprofloxacin and bacteria viability of *P. aeruginosa* biofilm after treatment with prodrug and nanozyme demonstrates comparable efficiency to that of the free drug. Reprinted from Ref. [184] with permission from the American Chemical Society. Copyright 2020

Bacterial biofilms are protected by an extracellular polymeric substance (EPS) composed of polysaccharides, DNA, lipids, and proteins that prevents the penetration of foreign species, such as drug molecules and radiation [243]. Rotello et al. developed a BONZ based on tetraphenyl porphyrin Fe(III) chloride (FeTPP) encapsulated into polyoxanorbornene polymeric NPs (PONI-C11-TMA) [184]. The polymer scaffold contained a positively charged trimethylamine headgroup, which facilitated the uptake of the nanozymes by bacteria cells (Fig. 28a–c). This "Trojan horse" strategy enabled nanozymes to penetrate the EPS, reach bacteria cells, and rapidly release the antimicrobial drugs, moxifloxacin and ciprofloxacin, from their azide-protected counterparts. The approach was effective against *P. aeruginosa* and *E. coli* biofilms, demonstrating a comparable efficiency to that of free drugs (Fig. 28d–g). Lastly, the nanozymes exhibited no acute cytotoxicity toward 3T3 fibroblasts, indicating their biocompatibility.

**Fig. 29:**
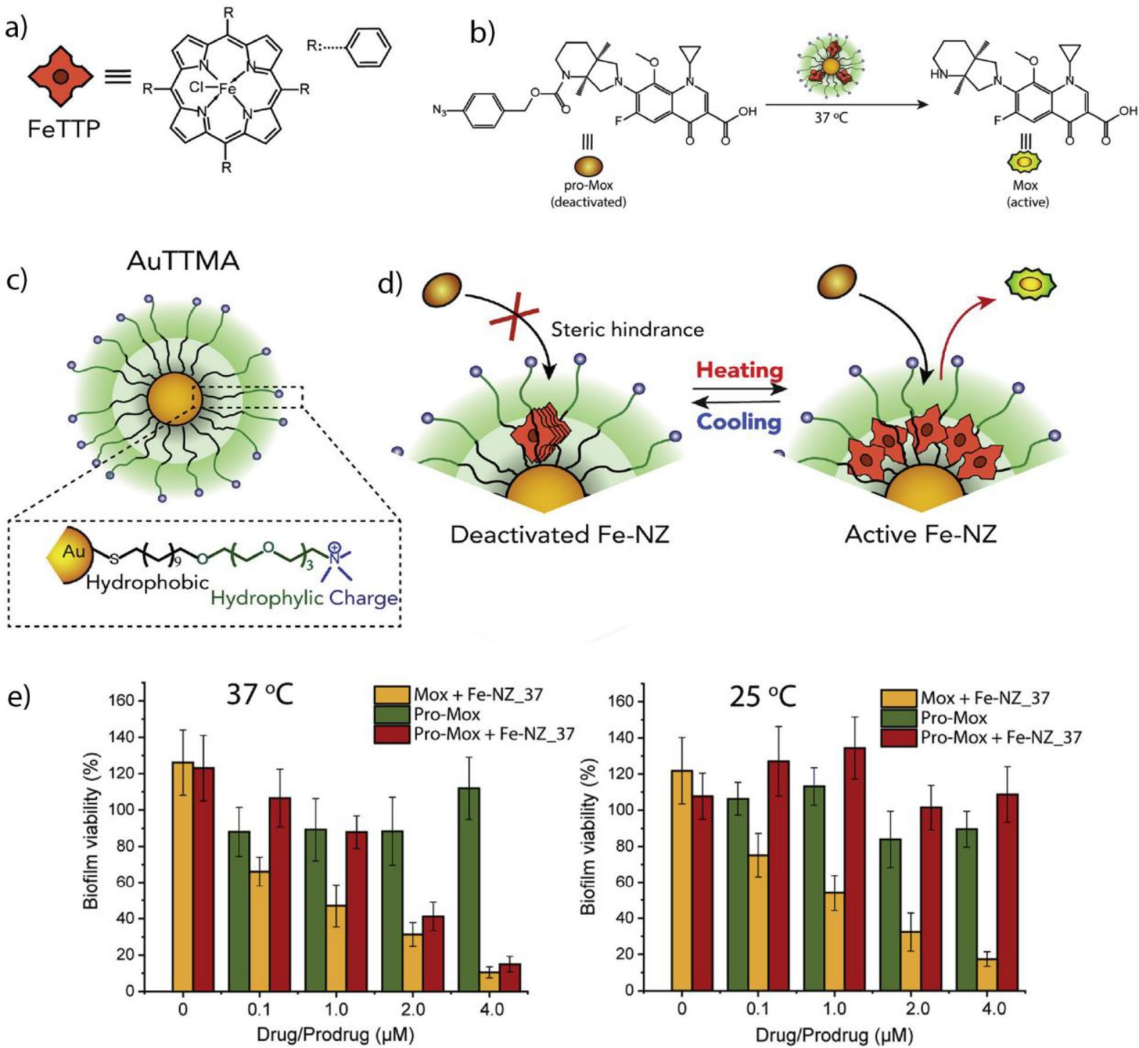
**a** Structure of FeTPP. **b** Bioorthogonal activation of the azide-protected moxifloxacin (pro-Mox) at 37 °C. **c** Structure of the organic ligand attached to Au NP core. **d** Mechanism of the temperature-controlled activation of nanozymes. **e** Therapeutic efficacy of nanozymes and prodrugs at 37 and 25 °C. Nanozyme exhibited negligible bacteria-killing activity at 25 °C and activity similar to that of the free drug at 37 °C. Reprinted from Ref. [204] with permission from Elsevier Inc. Copyright 2020

Rotello et al. also prepared thermally gated nanozymes by encapsulating FeTPP into Au NPs [204]. The Au NPs (2 nm) were decorated with an organic ligand, hydrophilic layer, and cationic trimethylamine headgroup, which formed a hydrophobic monolayer around the nanoparticle core, improved water solubility, and enabled intracellular uptake, respectively (Fig. 29a–c). Due to the extensive *π*-interactions of porphyrin-based metal complexes and the densely packed monolayer around the Au core, the encapsulated TMC demonstrated *π*–*π* stacking at room temperature, which reduced the catalytic activity. Upon reaching a certain temperature (changed in 3 °C increments), the heme stacking was disrupted; catalysis was restored (Fig. 29d). The nanozymes activated azide-protected moxifloxacin and were as effective as the free drug for treating *E. coli* at 37 ^◦^C but not at 25 ^◦^C (Fig. 29e).

**Fig. 30:**
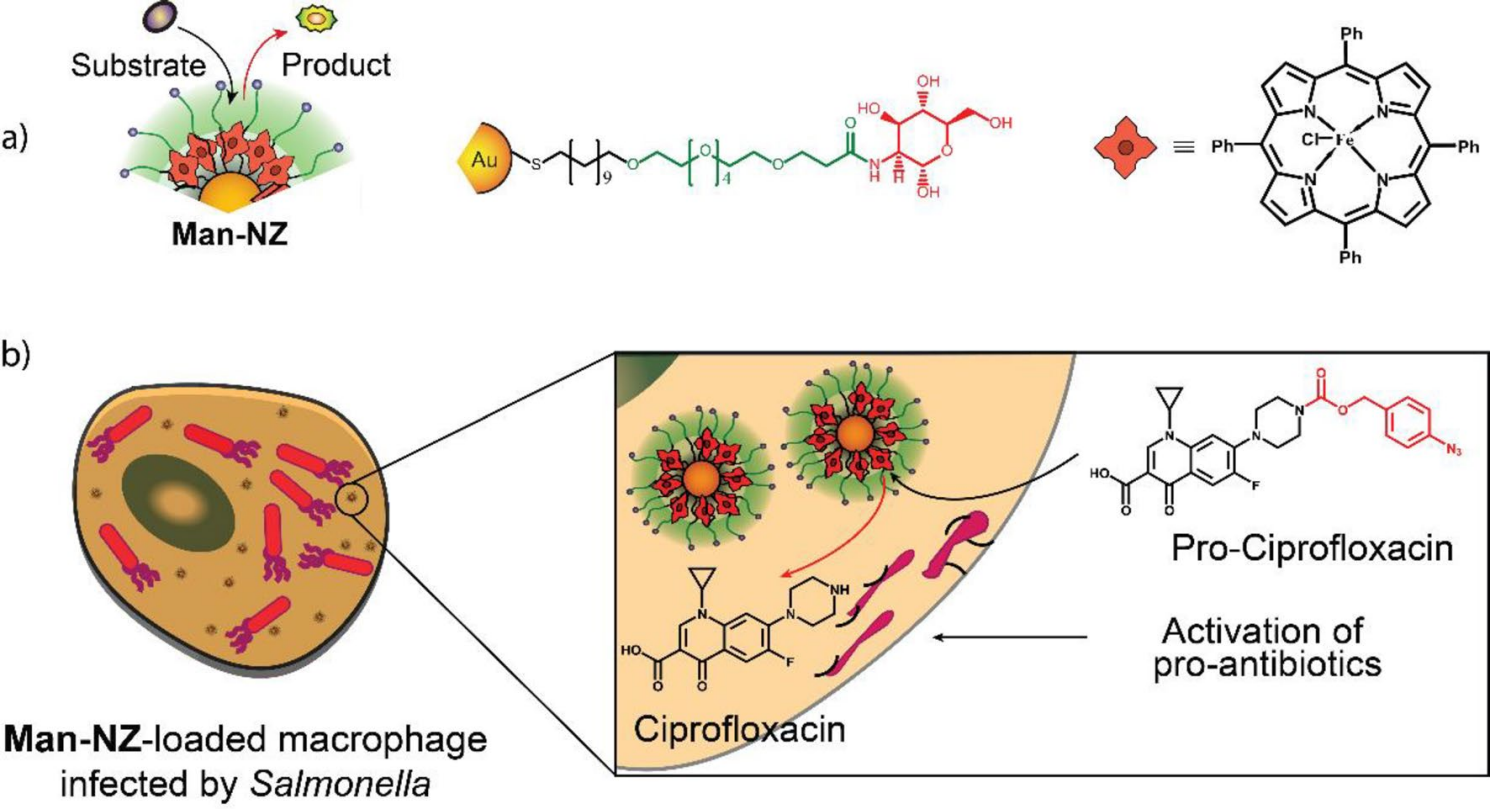
**a** Schematic of the substrate activation using nanozymes. The nanozyme was based on FeTPP encapsulated in Au NP decorated with an organic ligand and a mannose headgroup. **b** Targeting macrophages through the mannose moieties and activation of bioorthogonal catalysis to release azide-protected ciprofloxacin. Redrawn from Ref. [205]

Bacterial infections are not limited to wound biofilms. *Salmonella* causes systemic infections that cannot be treated easily using antimicrobial drugs due to the negative effect of these medicines on the human microbiome [205]. *Salmonella* evades the immune system and invades human macrophages, causing prolonged suffering in infected patients. The conjugation of the natural sugar mannose to the exterior of NPs allows the specific targeting of macrophages and intracellular uptake [244]. Rotello et al. efficiently targeted *Salmonella*-infected macrophages by conjugating mannose to the organic ligand of Au NPs and encapsulating FeTPP into the monolayer (Fig. 30a, b) [205]. These BONZs exhibited a considerable reduction in bacteria viability (more than 100-fold) after incubation with azide-protected ciprofloxacin compared to untreated control, suggesting their potential for cellular targeting and localized drug release.

### Au-based TMCs

Au(I)- and Au(III)-based TMCs can catalyze intramolecular cyclization and propargyl uncaging reactions by selectively activating alkyne bonds [206]. Au(I)-based TMCs possess a low cyto- and genotoxicity but Au(III) TMCs can coordinate to DNA, leading to errors in DNA replication similar to Pt (II) owing to the square-planar geometry of their complexes [245]. Moreover, Au(I) TMCs are vulnerable to free thiols owing to the potential formation of Au–S bonds, which may reduce the catalytic activity [246].

**Fig. 31:**
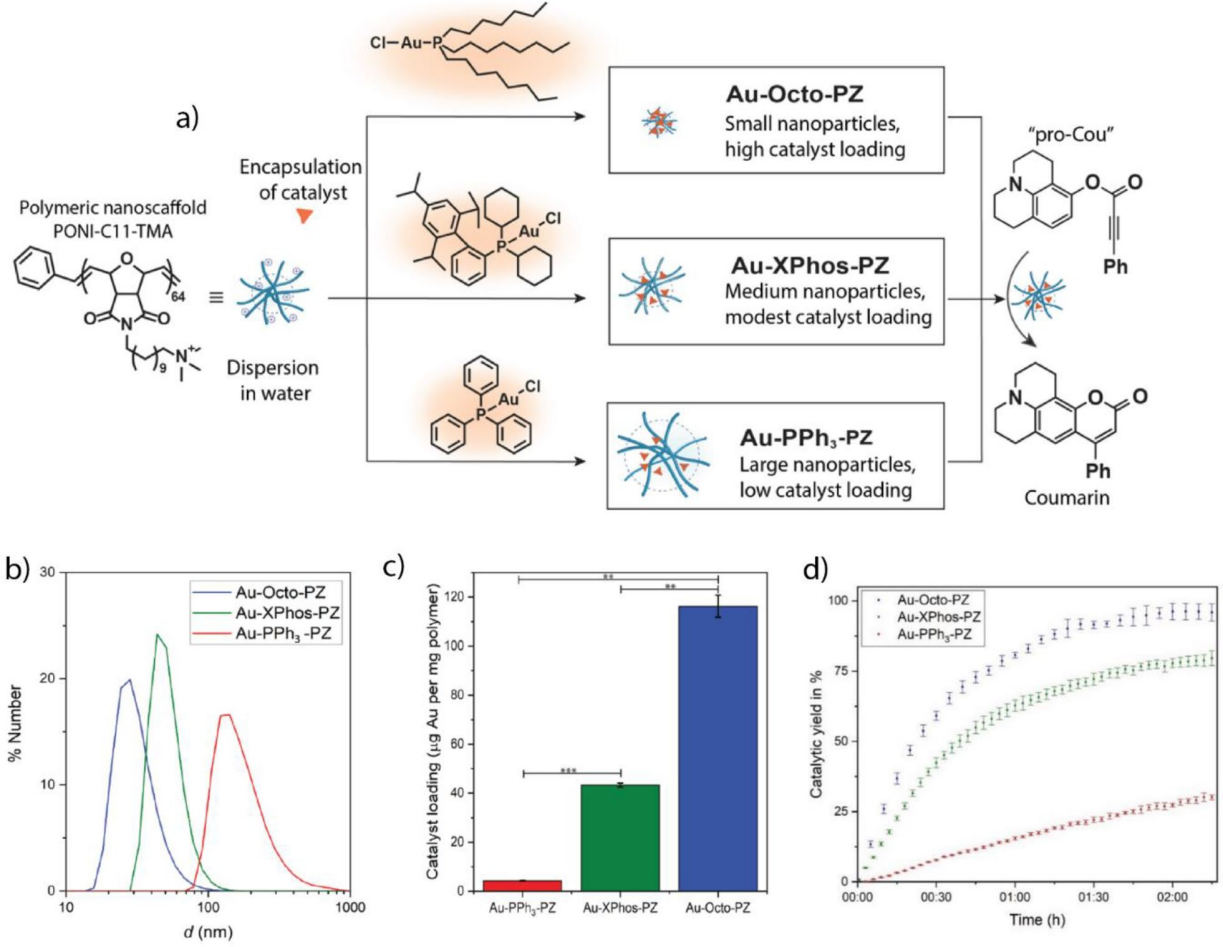
**a** Effect of catalyst choice on the formation and characteristics of nanozymes. **b** Size of fabricated nanozymes with different encapsulated Au-based TMCs. **c** Catalyst loading of the nanozymes depending on the Au TMC structure. **d** Effect of different Au TMCs on catalytic performance. Au-Octo-PZ had better activity compared to that of Au-XPhos- and Au-PPh_3_-PZ toward the cyclization of a coumarin derivative. Reprinted from Ref. [189] with permission from MDPI. Copyright 2022

Recently, novel protective groups cleavable by Au(I) complexes have been developed as advanced Au-based TMC nanozymes [231, 247]. For example, Rotello et al. designed Au(I) phosphine catalysts with different ligand backbones and encapsulated them into PONI-C11-TMA polymeric NPs. This BONZ can catalyze the cyclization of a non-fluorescent substrate into a fluorescent coumarin derivative (Fig. 31a) [189]. Depending on the ligand structure of the encapsulated organometal catalyst, the catalyst loading and overall catalytic performance increased (Fig. 31b–d). As such, the interactions between the nano scaffold and TMCs can be used to modulate bioorthogonal catalysis.

### Prospective bioorthogonal TMCs and reactions

Apart from the discussed TMCs and bioorthogonal reactions, catalysts based on other transition metals can be developed and used to fabricate nanozymes. For instance, Ir(I) complexes can catalyze allyl carbamate uncaging reactions intracellularly with a considerably lower cyto- and genotoxicity than the currently used Ru TMCs [248]. Furthermore, other bioorthogonal reactions can be performed with the currently available nanozymes, such as the Cu-catalyzed Sonogashira cross-coupling reaction for forming complex molecules or the Ru-catalyzed olefin metathesis and azide–thioalkyne cycloaddition [226, 249]. Other substrates for bioorthogonal transformations may be developed in the future to expand the possible applications of BONZs.

## Summary

As a new class of biocatalysts or artificial enzymes, nanozymes have received considerable attention owing to their immense potential in biomedical applications. Two approaches for utilizing nanozymes in intracellular chemistry have been reported. First, biomimetic nanozymes that exhibit properties similar to those of natural enzymes may be directly used for catalysis. Second, bioorthogonal catalysis may be employed through the integration of transition metal catalysts and nanomaterials. In both strategies, the proof of concept for catalytic functions and the potential biomedical applications of nanozymes have been reported. However, their practical use has been limited by some inherent drawbacks. To achieve comparable activity with those of natural enzymes, the catalytic mechanism using BMNZs at the molecular level should be fully understood. The catalyst should also be implemented and used based on mechanistic knowledge. Only a few families of enzymes have been mimicked, causing the limited diversity of BMNZs used in biomedical applications. The inherent toxicity of transition metals that provide active sites of BONZs should also be addressed. Nano scaffolds must be introduced to protect the metal catalysts. Both nanozymes encounter challenges in in vivo applications related to pharmacokinetics and pharmacodynamics.

The application of nanozymes to date has been primarily fundamental, with a primary focus on areas of cancer treatment, anti-bacterial, antioxidation, and biosensing. Their translation to the clinic will require overcoming challenges. For instance, although nanozymes show promise in cancer therapy, concerns regarding their biological safety remain. Therefore, systematic studies on the dynamics and eventual outcomes of nanozymes are essential, especially at various stages of treatment. These investigations will need to encompass pharmacokinetics, absorption, distribution, metabolism, duration of therapeutic effect, excretion, and nanozyme toxicity. Also, nanozymes (and all nanomaterials) tend to accumulate primarily in the liver, lung, and spleen. To improve therapeutic efficacy for diseases outside these organs it becomes crucial to confer targeting ability to nanozymes through surface modifications. Overall, nanozymes will need to overcome the same hurdles as small molecule and biologics therapeutics, clearly an accessible goal.

In the near future, natural enzyme mimetics may autonomously control their activities through conformational changes. Artificial zymogens may be activated through specific cleavage reactions. Various strategies to modify the in vitro or in vivo nanozyme activities will emerge, expanding their applications. As their activities approach those of natural enzymes, nanozymes offer a promising solution to address enzyme-related diseases and may provide unique advantages not found in natural enzymes.

## Data Availability

The review is based on the published data and sources of data upon which conclusions have been drawn can be found in the reference list.
